# Investigation of the Link between Per- and Polyfluoroalkyl Substances and Stress Biomarkers in Bottlenose Dolphins (*Tursiops truncatus*)

**DOI:** 10.1021/acs.est.3c06979

**Published:** 2024-05-14

**Authors:** Baylin J. Bennett, Max T. Aung, Rudy Boonstra, Brendan Delehanty, Magali Houde, Derek C. G. Muir, Patricia A. Fair, Matthew O. Gribble

**Affiliations:** † Gangarosa Department of Environmental Health, Rollins School of Public Health, Emory University, Atlanta, Georgia 30322, United States; ‡ Department of Population and Public Health Sciences, University of Southern California, Los Angeles, California 90032, United States; § Centre for the Neurobiology of Stress, Department of Biological Sciences, University of Toronto Scarborough, Toronto, Ontario M1C 1A4, Canada; ∥ Aquatic Contaminants Research Division, Environment and Climate Change Canada, Montreal, Quebec G1J 0C3, Canada; ⊥ Aquatic Contaminants Research Division, Environment and Climate Change Canada, Burlington, Ontario L7S 1A1, Canada; # Department of Public Health Sciences, Medical University of South Carolina, Charleston, South Carolina 29425, United States; ∇ Department of Medicine, Division of Occupational, Environmental and Climate Medicine, University of California San Francisco, San Francisco, California 94143, United States

**Keywords:** environmental pollutants, cetacean health, wildlife biomonitoring, ecotoxicology, marine biology, one health, bottlenose dolphin

## Abstract

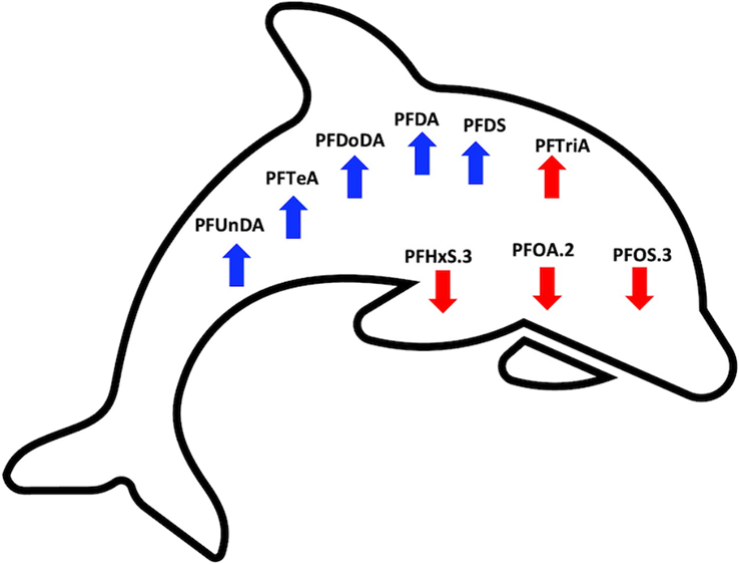

Bottlenose dolphins (*Tursiops truncatus*) are keystone and sentinel species in the world’s oceans. We studied correlations between per- and polyfluoroalkyl substances (PFAS) and their stress axis. We investigated associations between plasma biomarkers of 12 different PFAS variants and three cortisol pools (total, bound, and free) in wild *T. truncatus* from estuarine waters of Charleston, South Carolina (*n* = 115) and Indian River Lagoon, Florida (*n* = 178) from 2003 to 2006, 2010–2013, and 2015. All PFAS and total cortisol levels for these dolphins were previously reported; bound cortisol levels and free cortisol calculations have not been previously reported. We tested null hypotheses that levels of each PFAS were not correlated with those of each cortisol pool. Free cortisol levels were lower when PFOS, PFOA, and PFHxS biomarker levels were higher, but free cortisol levels were higher when PFTriA was higher. Bound cortisol levels were higher when there were higher PFDA, PFDoDA, PFDS, PFTeA, and PFUnDA biomarkers. Total cortisol was higher when PFOA was lower, but total cortisol was higher when PFDA, PFDoDA, PFTeA, and PFTriA were higher. Additional analyses indicated sex and age trends, as well as heterogeneity of effects from the covariates carbon chain length and PFAS class. Although this is a cross-sectional observational study and, therefore, could reflect cortisol impacts on PFAS toxicokinetics, these correlations are suggestive that PFAS impacts the stress axis in *T. truncatus*. However, if PFAS do impact the stress axis of dolphins, it is specific to the chemical structure, and could affect the individual pools of cortisol differently. It is critical to conduct long-term studies on these dolphins and to compare them to populations that have no or little expose to PFAS.

## Abstract 2

Our results are the first to report associations between blood plasma PFAS levels and the different pools of blood cortisol in wild *T. truncatus*.

## Introduction

Environmental contaminants may cause adverse effects in organisms. These impacts can be understood by examining an organism’s stress axis, which multitasks throughout their life^1^ and is pivotal for successful adaptation, within and between species, in four ways. First, the stress axis is involved in normal diurnal cycle and activities such as exploratory and food-seeking behaviors (reviewed in refs (2) and (3)). Second, the axis responds with short-term physiological adjustment to maintain survival during acute, environmental stressors (i.e., “flight or fight” reaction).^4^ Chronic or permanent exposure to environmental stressors may also alter the stress axis, physiology, and reproduction of the animal. Further, the limbic system and the hypothalamic-pituitary-adrenal (HPA) axis are only one part of the stress response, which includes other hormones, neurotransmitters, opioid peptides, cytokines, and brain functions.^5^ Third, the stress axis can be permanently programmed during embryonic development by stressors affecting the mother; this may lead to the adaption of the individual to new conditions it experiences during its lifetime.^6^ Fourth, it is subject to evolutionary modification and equips species to succeed under different ecological roles and contexts. The impact of pollution on the stress axis is an understudied field.

Cortisol is an established biomarker of the stress axis in wildlife^7,8^ and its levels can be influenced by external contaminants and other factors. Although not the only key player, nor the only system it functions in, cortisol plays an important role in an organism’s stress axis. Typically, corticosteroid-binding globulin (CBG) binds 85–90% of cortisol. Cortisol is released from CBG in response to a stimulus,^9,10^ which allows the body to return to a state of rest. This cortisol-CBG complex is known as the bound cortisol fraction. The remaining unbound cortisol is termed free cortisol. Free cortisol can leave the bloodstream and have biological activity such as increasing heartbeat rate and blood glucose levels.^11,12^ Although not biologically active, bound cortisol is still biologically relevant due to its theorized role as a cortisol reservoir.^9^ Essentially, total cortisol (bound fraction + free fraction) circulates the body until the free fraction enters tissues via capillaries and elicits effects modulated by corresponding cortisol receptors. Once the free fraction leaves circulation, bound cortisol unbinds thereby replenishing the free fraction. Bound cortisol, therefore, is biologically relevant because it serves a functional role, but is not active since it cannot have a direct impact on tissues. Under conditions of prolonged and chronic stress, an increase in total cortisol levels occurs. Chronic total cortisol activity can cause a decline in CBG binding capacity in mammals and birds.^9^ This directly impacts vital survival functions for vertebrates, such as the suppression of reproductive processes,^13,14^ as well as have negative individual health outcomes like a decrease in immunocompetency^15^ and, consequently, an increase in vulnerability and susceptibility to diseases.^16^

Per- and polyfluoroalkyl substances (PFAS) are humanmade chemicals used in everyday products, such as in nonstick cookware, weather-resistant apparel, and fire-suppressing foams.^17^ The PFAS family comprises over 10,000 chemicals.^18^ They can bioaccumulate and biomagnify in the environment and wildlife^19−21^ allowing for their global detection in the soil, waterways, and atmosphere.^22−25^ Exposure to two long-chain perfluoroalkyl acids (PFAAs), perfluorooctanesulfonic acid (PFOS) and perfluorooctanoic acid (PFOA), can cause liver damage, pre-eclampsia, and other negative health outcomes.^26^ PFOS was added to the Stockholm Convention’s list of *Persistent Organic Pollutants to Avoid* in 2009,^27^ and PFOA was added in 2017.^28^ This boosted involvement in the ongoing voluntary phaseout, which started in 2006,^29^ where manufacturers switched to alternative short-chain PFAS. The rationale was that these PFAS were safer (i.e., less bioaccumulative) than long-chain predecessors;^30^ however, some short-chain PFAS were reported to be more environmentally mobile and persistent.^19,31^ Environmental PFAS, like other contaminants, exists as individual exposures and chemical mixtures. The full impact of PFAS may have detrimental effects at each level of the stress axis and investigations should more fully explore these.

To our knowledge, no wildlife study has assessed pollution impacts on the three cortisol pools–total, bound, and free–independently. Given the apparent relationship between environmental pollutants and stress hormones, this study aimed to assess associations between individual PFAS biomarkers and individual cortisol pool levels in plasma of wild *T. truncatus* populations from the estuarine waters of Charleston, South Carolina and Indian River Lagoon, Florida, USA.

## Materials and Methods

### Study Description

Samples were collected in 2003–2006, 2010–2013, and 2015 as part of the Bottlenose Dolphin Health and Risk Assessment (HERA) Project which examined relationships between health and environmental conditions in two bottlenose dolphin populations along the eastern coast of the United States: Indian River Lagoon, Florida, and the estuarine waters of Charleston, South Carolina.^22,32^ Free-ranging dolphins were temporarily restrained and physically examined; blood and tissue samples were collected as previously described in detail.^33^ Briefly, due to direct relationship between cortisol levels and time during capture-and-release, fluke-vein blood samples were collected immediately after disentanglement from wild, free-range bottlenose dolphins off the coast of Charleston, South Carolina (*n* = 115) and Indian River Lagoon, Florida (*n* = 178). Samples were immediately centrifuged postcollection and the plasma was transferred to cryovials, which were stored at −20 °C until analysis. Samples from dolphins were collected under National Marine Fisheries Permit nos. 998-1678 and 14352-03 issued to Dr. Gregory Bossart and approved by the Florida Atlantic IACUC under Protocol #A10-18. Sex was determined at capture; age was estimated using an extracted tooth to examine the dentine layers. Table S1 lists the sample sizes for location, age, and sex by sample collection year. Details regarding the HERA Project dolphin cohorts have been previously published summarizing the demography, health characteristics, and environmental exposures including both chemical pollutants and microbial agents.^22,32,34,35^

### Blood Cortisol Pool Separation and Measurement

We measured the three pools of cortisol–total, CBG-bound, and free–in these dolphins. Fair et al.^7^ reports the measurement method and its validation to quantify total cortisol levels in these dolphins. Delehanty et al.^8^ reports the measurement method and its validation to quantify CBG-bound cortisol and from this calculated the free cortisol. Here we lay out briefly how this was done on these dolphins for our study. Triglycerides were saponified using NH_4_OH; the aqueous layer was aspirated and then evaporated in filtered air. Bound and free cortisol were separated from the samples using dextran-coated charcoal and then measured using a scintillation counter.

Bound cortisol consists of both corticosteroid-binding globulin bound cortisol and nonspecifically bound cortisol (bound loosely by other plasma proteins, primarily albumin). For this study, nonspecifically bound cortisol was considered free, and only CBG-bound cortisol was considered to make up the bound pool of cortisol. CBG binding capacity was measured using previously described methods.^36^ Briefly, two separate aliquots of plasma were used: one to measure total binding and the other to measure nonspecific binding. To the first aliquot, tritiated cortisol was added. To the second aliquot, a combination of the tritiated cortisol and an excess of unlabeled cortisol were added. The desired CBG binding capacity of the plasma was calculated by subtracting the scintillation counts per minute (cpm) of the second aliquot (nonspecific binding) from the cpm of the first aliquot (total binding).

Free cortisol cannot be easily measured directly, so it was calculated using a previously established equation.^8,36,37^ Briefly, the three values used for the calculation are the concentration of the total cortisol, the species-specific binding affinity (*K*_d_) of CBG (2.6 nM at 37 °C for BND:,^8^ and the measured CBG binding capacity. For this study we considered the cortisol pools as existing individually.

### PFAS Extraction and Measurement

The plasma PFAS analytical method for *T. truncatus* and results were previously published.^35,38,39^ Twelve PFAS were individually isolated and measured: PFDA, PFDS, PFDoDA, PFHpA, PFHxS, PFNA, PFOA, PFOS, PFOSA, PFTeA, PFTriA, and PFUnDA (see Table S2 for full names and limits of detection by year). Further, for the purpose of these analyses, they were also considered in two separate groups: total perfluoroalkyl carboxylates (ΣPFCA = PFDA, PFDoDA, PFNA, PFOA, PFHpA, PFTeA, PFTriA, and PFUnDA) and total perfluoroalkyl sulfonates (ΣPFSA = PFDS, PFHxS, and PFOS, as well as the sulfonamide, PFOSA). Total PFAS (ΣPFAS) was considered as well. See Table 1 for a detailed example that lists the values for the mean, 25th percentile, median, 75th percentile, and interquartile range (IQR) for each cortisol pool within each PFOS tertile.

**Table 1:** Values for Mean, 25th Percentile, Median, 75th Percentile, and IQR for Each Cortisol (10 nM) Pool within Each PFOS Tertile

**tertile**	**cortisol (10 nM) pool**	**mean**	**25th percentile**	**median**	**75th percentile**	**IQR**
1	free	3.03	2.00	2.89	3.92	1.93
	bound	4.47	1.83	3.64	6.23	4.41
	total	7.02	4.44	6.59	8.83	4.39
2	free	2.86	1.51	2.64	4.01	2.50
	bound	4.02	1.98	3.81	5.61	3.64
	total	6.48	4.41	6.43	8.32	3.90
3	free	2.56	1.66	2.58	3.30	1.64
	bound	3.52	2.13	3.63	4.70	2.57
	total	5.70	4.28	5.63	7.23	2.95
total	free	2.82	1.70	2.64	3.70	2.01
	bound	4.00	2.00	3.73	5.50	3.50
	total	6.41	4.39	6.07	8.11	3.72

### Descriptive Analysis

Principal components analysis (PCA) was performed on log transformed PFAS levels due to their skewed nature (i.e., biomarker concentrations were skewed when not transformed and had more normal distributions after log-transformation). When PFAS levels were below their respective limit of detection (LOD), they were replaced with their LOD prior to being log-transformed (see Table S2 for specific LODs). The scores from PCA1 were then separately assessed for associations with the three pools of cortisol using linear regression.

### Statistical Analysis

Blood cortisol (total, bound, or free) was used as the dependent variable in a proportionate percentiles parametric quantile regression model with Huber–White robust standard errors.^40,41^ PFAS exposures were modeled as tertiles (See Table S3 for PFAS tertile sample sizes, means, and cutoff ranges). Models were adjusted for dolphin age, sex, sampling year, and location at time of sample collection.^35^ The generalized gamma distribution is a three-parameter generalization of the gamma distribution which includes many skewed distributions, including the log-normal distribution and the Weibull distribution, as special cases.^42^ First, full proportionate percentiles parametric quantile regression models were fit assuming a generalized gamma distribution and evaluated if the maximum likelihood estimate for the shape parameter (kappa) was significantly different from 0 or from 1. If the maximum likelihood generalized gamma was consistent with either log-normal or Weibull distribution, we fitted a simplified model assuming the appropriate distribution. If both distributions fit, we used Weibull. More specifically, Weibull distribution was assumed for all PFAS in the models containing total cortisol. Additionally, Weibull distribution was assumed for all PFAS in the models containing free cortisol, except for PFHpA and PFDS in which log-normal was assumed for both models. For all PFAS in the models containing bound cortisol, log-normal distribution was assumed. A full list of model distributions can be found in Table S4.

For descriptive secondary analyses, all unadjusted models used the chosen distributions for the respective adjusted, nonstratified models. For example, since the distribution for the full model evaluating associations between free cortisol and tertiles of PFOS assumed Weibull distribution, all other models evaluating associations between free cortisol and tertiles of PFOS (i.e., the unadjusted model, the unadjusted and stratified model, and the full and stratified model) assumed a Weibull distribution as well.

For secondary analyses, two independent methods were conducted: 1) stratification and 2) meta-regression. For the first method, the models were stratified separately by sex (male or female) or age (juvenile or adult). A wide variety of age categories have been utilized for classifying sexual maturity in bottlenose dolphins; female ages range from 5 to 12 years old and males from 10 to 13 years.^43^ For the age categories examined in this study, juveniles were considered as females <7 years old and males <10 years old, and adults were considered as females ≥7 years old and males ≥10 years old. The data set contained an age range from 2.5 years old to 33 years old. For the second method, we tested the heterogeneity of effects according to covariates using inverse-variance weighted mixed-effects meta-regression. There were two covariates assessed: carbon chain length and PFAS class, which were tested in separate models (see Table 3 for results). We included all regression coefficients (i.e., tertile 2 versus 1 and tertile 3 versus 1) in the models. Meta-regression models were fitted separately for free, bound, and total cortisol. Carbon chain length was coded as a binary variable for < 10 carbons (PFHpA, PFHxS, PFNA, PFOA, PFOS, and PFOSA) or ≥ 10 carbons (PFDA, PFDoDA, PFDS, PFTA, PFTriA, and PFUnDA). Additionally, the nonstratified models' type I error rate control was considered separately. The type I error rate control threshold assuming 450 independent statistical tests (all models) was α = 0.0001. Among the 90 independent statistical tests (nonstratified models only), the type I error rate control threshold was α = 0.0006.

Finally, all *p*-values presented were from Z-tests. Stata 16.1 IC software was used to perform all statistical analyses.

## Results

The median (interquartile range) age was 8 years old (17–4) for females and 13 years old (18–9) for males. The median (IQR) concentrations for each cortisol pool were as follows: total = 58.21 nM (78.07–40), bound = 26.91 nM (38.21–17.02), and free = 33.06 nM (51.28–16.01). Table 2 lists the median (IQR) concentrations for each PFAS. More detailed descriptive analyses of these dolphins, cortisol biomarkers, other stress axis biomarkers (i.e., adrenocorticotropin hormone, and aldosterone), other hormones (i.e., epinephrine, norepinephrine, dopamine), and PFAS have already been published.^7,22,38,39^

**Table 2:** Median and IQR Concentrations (ng/g) for PFAS

**PFAS median and IQR concentrations**		
**PFAS**	**median** (ng/g)	**IQR** (ng/g)
PFDA	19.6	90.6–7.7
PFDS	2.3	10.0–1.2
PFDoDA	2.4	6.4–1.2
PFHpA	0.9	1.7–0.8
PFHxS	21.8	62.8–11.3
PFNA	15.9	57.9–7.9
PFOA	10.8	30.0–4.7
PFOS	696.1	1388.5–330.3
PFOSA	4.4	22.4–0.5
PFTeA	0.4	1.7–0.5
PFTriA	1.6	3.1–0.4
PFUnDA	12.8	49.1–7.0
ΣPFCA	141.2	304.4–45.6
ΣPFSA	957.0	1626.9–434.4
ΣPFAS	941.8	1780.7–388.6

The results for the principal components analysis (PCA) are listed in Table S5. PCA1 (eigenvalue: 8.63) accounted for 78.5% of the variance, PCA2 (0.89) accounted for an additional 8.1%, and PCA3 (0.64) accounted for a further 5.8% for a total of 92.4% of the variance. The loadings in PCA1 were all positive (i.e., the score increases with higher concentrations of each PFAS) whereas there were no intelligible patterns in the loading directions in PCA2 and PCA3; therefore, we assessed the associations of PCA1 scores with the different pools of cortisol. The associations between PCA1 scores and the pools of cortisol were 0.95 (95% confidence interval: 0.73,1.24; *p* = 0.720; *n* = 28) for free cortisol, 0.86 (95% CI: 0.75,0.97; *p* = 0.019; *n* = 29) for bound cortisol, and 0.88 (95% CI: 0.62,1.25; *p* = 0.466; *n* = 28) for total cortisol.

### Type I Error Rate Control

**Figure 1:**
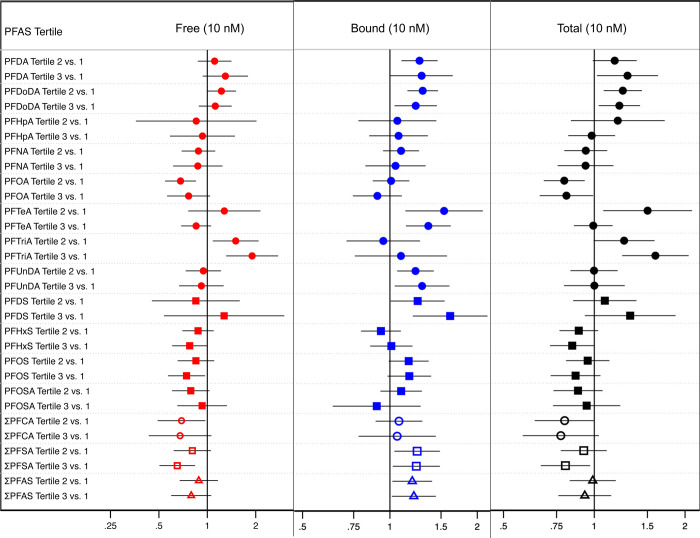
Nonstratified results for adjusted parametric quantile regression models testing for percent differences in cortisol (10 nM) across PFAS tertiles. Models were adjusted for location, year, sex, and age at time of sample collection. Shapes indicate which class the modeled PFAS belongs to a filled circle = PFCA class, a filled square = PFSA class and an empty shape = the sum of a single class or both classes combined (i.e., empty circle = ΣPFCA, empty square = ΣPFSA, and empty triangle = Σall PFAS combined). Regression coefficients, 95% confidence intervals, and *n* for each model is listed in Table S6.

Figure 1 lists results for all nonstratified models (see Table S6 for quantile ratios, 95% confidence intervals, and model sample sizes for all adjusted models). We considered two Bonferroni significance thresholds: a strict threshold of *p* = 0.0001 which included the sex- and age-stratified models in the total test number (*n* = 450 tests) and a more relaxed threshold of *p* = 0.0006 which did not include the stratified models (*n* = 90 tests). Only one association was considered Bonferroni-significant at α = 0.0001: the nonstratified model assessing bound cortisol and the second tertile of PFDoDA exposure (*p* < 0.0001). When considering a Bonferroni-significant alpha threshold change from *p* = 0.0001 to *p* = 0.0006, in addition to the above association, only one other association was Bonferroni-significant: the nonstratified model assessing total cortisol and the third tertile of PFTriA exposure (*p* = 0.0003).

### Meta-Regression

Free cortisol results showed effect heterogeneity from carbon chain length and PFAS class. The average association with free cortisol for being in PFAS tertile 2 or 3 versus tertile 1 was more negative for shorter-chain (< 10 carbons) PFAS than for longer-chain (≥ 10 carbons) PFAS. Similarly, the average association with free cortisol for being in tertile 2 or 3 versus tertile 1 for PFSAs was more negative than for PFCAs. Bound cortisol showed effect heterogeneity from carbon chain length and PFAS class. The average association for bound cortisol of being in tertile 2 or 3 vs. tertile 1 was more positive for longer-chain (≥ 10 carbons) PFAS than for shorter-chain (< 10 carbons) PFAS. Likewise, the average association between being in tertile 2 or 3 vs. tertile 1 for PFSAs with bound cortisol was more positive than for PFCAs (see Table 3 for results). None of these covariates showed effect heterogeneity for total cortisol.

**Table 3:** Meta-Regression Models Testing the Covariates Carbon Chain Length and PFAS Class (Tertile 2 or 3 Versus 1) with Different Cortisol Pools. Meta-regression findings are presented as the ratio of the measures of association [i.e., the quantile ratio of cortisol (free, bound, or total) for greater than 1st tertile vs. 1st tertile (i.e., pooling 2nd and 3rd tertile effects)], among one level of the tested covariate vs. the other level of the tested covariate.

**Associations of covariates with the estimated PFAS-cortisol pool associations**				
**covariate**	**cortisol pool**	**quantile ratio ratio**	**lower 95% CI**	**upper 95% CI**
carbon chain length (≥ 10 versus < 10 carbons)	free	0.88	0.78	0.97
	bound	1.08	1.03	1.13
	total	0.95	0.89	1.02
PFAS class (PFSA versus PFCA)	free	0.83	0.70	0.97
	bound	1.11	1.01	1.21
	total	0.94	0.83	1.05

### Sex and Age Stratification

**Figure 2:**
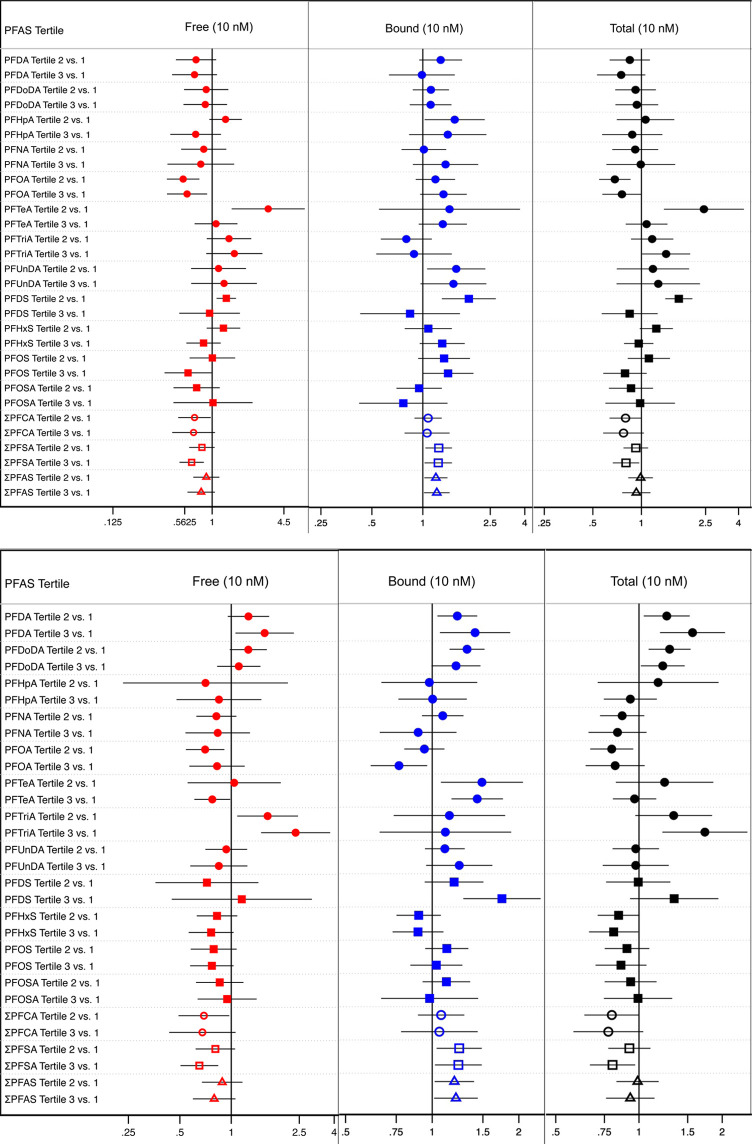
Sex-stratified results for female-stratified (top) and male-stratified (bottom) adjusted parametric quantile regression models testing for percent differences in cortisol (10 nM) across PFAS tertiles. Models were adjusted for location, year, sex, and age at time of sample collection. Shapes indicate which class the modeled PFAS belongs to a filled circle = PFCA class, a filled square = PFSA class and an empty shape = the sum of a single class or both classes combined (i.e., empty circle = ΣPFCA, empty square = ΣPFSA, and empty triangle = Σall PFAS combined). Regression coefficients, 95% confidence intervals, and *n* for each model is listed in Table S6.

The statistically significant associations seen in the nonstratified model results were assessed for effect heterogeneity, noted as trends, when stratified by sex. Stratification by sex produced uneven sample sizes, where the sample sizes of the female dolphins were noticeably small, which yielded wider confidence intervals, compared to those of the male dolphins (Figure 2; see Table S6 for quantile ratios, 95% confidence intervals, and model sample sizes for all adjusted models).

Overall, the association trends between free, bound, or total cortisol and the individual PFAS were consistent upon stratification by sex; however, four unique trends appeared. First, female dolphins in the middle tertile of the PFHxS exposure trended positively with free cortisol, which was contrary to the nonstratified findings; male dolphins in both tertiles of the PFHxS exposure, as well as the female dolphins in the highest tertile of this exposure, trended negatively with free cortisol. Second, female dolphins in the highest tertile of the PFDS exposure trended negatively with bound cortisol, which was contrary to the nonstratified findings; male dolphins in both tertiles of the PFDS exposure, as well as the female dolphins in the middle tertile of this exposure, trended positively with bound cortisol. Third, female dolphins in the highest tertile of the PFDA exposure trended negatively with total cortisol, which was contrary to the nonstratified findings; male dolphins in the highest tertile of the PFDA exposure trended positively with total cortisol. Fourth, female dolphins in both tertiles of the PFDoDA exposure trended negatively with total cortisol, which was contrary to the nonstratified findings; male dolphins in both tertiles of the PFDoDA exposure trended positively with bound cortisol.

**Figure 3:**
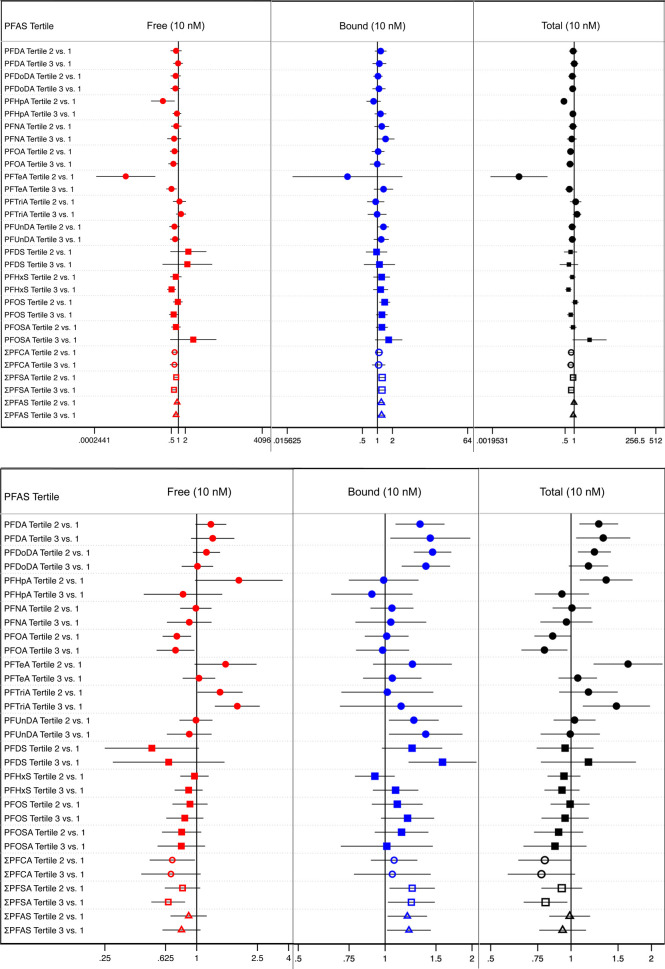
Age-stratified results for juvenile-stratified (top) and adult-stratified (bottom) adjusted parametric quantile regression models testing for percent differences in cortisol (10 nM) across PFAS tertiles. Juveniles were defined as female dolphins <7 years old and male dolphins <10 years old. Adult dolphins were defined as female dolphins ≥7 years old and male dolphins ≥10 years old. Models were adjusted for location, year, sex, and age at time of sample collection. Shapes indicate which class the modeled PFAS belongs to a filled circle = PFCA class, a filled square = PFSA class and an empty shape = the sum of a single class or both classes combined (i.e., empty circle = ΣPFCA, empty square = ΣPFSA, and empty triangle = Σall PFAS combined). Regression coefficients, 95% confidence intervals, and *n* for each model is listed in Table S6.

The statistically significant associations seen in the nonstratified model results were also assessed for effect heterogeneity, noted as trends, when stratified by age. Stratification by age produced uneven sample sizes, where the sample sizes of the juvenile dolphins were noticeably small, which yielded wider confidence intervals, compared to those of the adult dolphins (Figure 3; see Table S6 for quantile ratios, 95% confidence intervals, and model sample sizes for all adjusted models).

The association trends between free, bound, or total cortisol and the individual PFAS were consistent upon stratification by age. Several trends in the juvenile dolphins appeared to contradict the nonstratified results. However, closer inspection revealed the sample size of the juvenile dolphins in the respective PFAS tertiles was one, which led us to disregard the potential for this trend to contradict the original findings. Results for all the unadjusted models are listed as Table S7.

## Discussion

Our key conclusion is that the direction of PFAS-cortisol associations primarily differ by cortisol pool; PFAS-bound cortisol associations were consistently directly related, whereas both PFAS-free cortisol associations and PFAS-total cortisol associations varied. Further, age- and sex-stratified results for each PFAS-cortisol association mostly trended in consistent directions as their nonstratified counterparts. Interestingly, when the sex-stratified trends diverged from the nonstratified findings, it was the female-stratified results that differed. This could be the result of data sparsity, a limitation mentioned later, a sex-specific physiological phenomenon (i.e., offloading, bleeding during birth, or nursing), or another unknown phenomenon. Due to data sparsity, we were unable to assess whether age further influenced these varying trends. Strangely, the results from the Principal Component Analysis suggested that most variability increases with lower concentrations of PFAS; however, this pattern does not match the data likely because it is contextually incoherent with environmental fate and transport processes (e.g., we cannot accurately account for the origination of each PFAS pollutant).

We also found that the associations of higher PFAS concentrations (i.e., tertile 2 or 3 versus 1) with free cortisol and with bound cortisol had a stronger dose response with greater chain length. The fact that longer carbon chain PFAS, as well as PFSAs versus PFCAs, are known to have a higher binding affinity with serum-binding proteins^44^ may help explain why longer-chain compounds have stronger associations of elevated PFAS levels with lower free cortisol and higher bound cortisol levels.

Our study is not without caveats that need to be addressed since the scientific inferences from this observational ecotoxicology study are a function of both data and models. First, there is potential for data sparsity (e.g., rare combinations of variables in the data set) to influence model estimation and inference. Second, there is potential for the dolphins observed in this study to be not fully representative of other dolphins. Third, this is a cross-sectional study, thus, it is not possible to infer “cause” and “effect”; however, it is suggestive and warrants additional research. Fourth, other stress-response hormones and additional time points could offer further insight into possible mechanisms of the relationship between environmental PFAS contamination and the HPA in wildlife and humans. Fifth, there is also potential for measurement error from other influences on time-of-sampling cortisol besides PFAS to affect our estimated associations between PFAS and cortisol pools: although each dolphin in this study was handled in the same manner under the sample collection protocol, it is important to note that cortisol has been shown to increase in dolphins during capture-release health assessment studies.^7^ Sixth, this study used data from single samples rather than duplicates or triplicates collected from multiple cross-sectional studies, which could introduce measurement error. Finally, within the environment PFAS exist in mixtures with other PFAS and with other chemicals. Further, it has been established that the Bottlenose Dolphin HERA Project cohorts have been exposed to many other contaminants and microbial agents.^32,34^ Possible associations between PFAS mixtures and cortisol levels should be explored.

### Future Directions

Understanding the mobility, presence, and impact of PFAS is vital since they pose a significant threat to ecosystems globally. Our study highlights a major gap in the literature since environmental pollution-wildlife cortisol relationships are not considered in light of the different pools of cortisol. This gap is important to bridge since the cortisol pools play different physiological roles. Identifying mechanisms of the physiological impacts of PFAS have recently become popular.^45−47^ We urge future studies to consider impacts to the whole of the stress axis, especially the different cortisol pools, as well as conduct long-term studies. Taken together, these concepts are key to the preservation and future health of all ecosystems and their inhabitants.

## Supporting Information Available

Table S1. Sample sizes for location, sex, and age by years sampled. Table S2. Individual PFAS detection limits (ng/g wet weight) by year. Table S3. PFAS tertile sample sizes, means (ng/g), and cutoff ranges. Table S4. Distributions for each PFAS-cortisol model. Table S5. Principal component analysis results. Table S6. Regression coefficients, 95% confidence intervals, and sample sizes (*n*) for each adjusted model. Table S7. Regression coefficients, 95% confidence intervals, and sample sizes (*n*) for each unadjusted model (PDF)
