# Supplementary material for: Investigation of the Link between Per- and Polyfluoroalkyl Substances and Stress Biomarkers in Bottlenose Dolphins (Tursiops truncatus)
Source: Environ Sci Technol. 2024 May 14;58(21):9061–70. doi: 10.1021/acs.est.3c06979 (PMC11137861; doi:10.1021/acs.est.3c06979)
Supplement: Supplementary file 1 — es3c06979_si_001.pdf [file es3c06979_si_001.pdf]

Investigation of the Link between Per- and Polyfluoroalkyl Substances (PFAS) and Stress Biomarkers in Bottlenose Dolphins (*Tursiops truncatus*)

Baylin J. Bennett [ORCID: 0000-0001-6541-7868][1,7*], Max T. Aung [ORCID: 0000-0001-5541-5447][2], Rudy Boonstra [ORCID: 0000-0003-1959-1077][3], Brendan Delehanty [ORCID: 0000-0002-6104-9049][3], Magali Houde [ORCID: 0000-0001-5013-6529][4], Derek C. sG. Muir [ORCID: 0000-0001-6631-9776][5], Patricia A. Fair [ORCID: 0000-0002-1431-7880][6], and Matthew O. Gribble [ORCID: 0000-0002-1614-2981][7]

Table S1…..………………………………………………………………………………...S2
Table S2……..……………………………………………………………………………...S3
Table S3………..…………………………………………………………………………...S4
Table S4…………..………………………………………………………………………...S5
Table S5……………..……………………………………………………………………...S6
Table S6……..……..…………………………………………………………………S7 – S21
Table S7……………...……………………………………………………………...S21 – S35

Table S1. Sample sizes for location, sex, and age by years sampled.

	n by year									
	**2003**	**2004**	**2005**	**2006**	**2010**	**2011**	**2012**	**2013**	**2015**	**Total**
IRL, FL	57	35	14	7	17	13	19	0	16	178
Charleston, SC	59	19	18	0	0	0	0	19	0	115
Juvenile	32	21	13	2	2	2	0	1	2	75
Adult	84	33	19	5	15	11	15	15	14	211
Male	78	28	19	7	16	12	9	10	15	194
Female	38	26	13	0	1	1	6	6	1	92

Table S2. Individual PFAS detection limits (ng/g wet weight) by year.

		PFAS Limits of Detection by Year [1]								
**Abbreviation**	**Full Name**	**2003**	**2004**	**2005**	**2006**	**2010**	**2011**	**2012**	**2013**	**2015**
PFDA	Perfluorodecanoic Acid	0.800	0.800	0.800	0.228	0.228	0.228	0.039	0.039	0.228
PFDS	Perfluorodecane Sulfonic Acid	0.800	0.800	0.800	0.275	0.275	0.275	0.005	0.005	0.275
PFDoDA	Perfluorododecanoic Acid	0.500	0.500	0.500	0.433	0.433	0.433	0.005	0.005	0.433
PFHpA	Perfluoroheptanoic Acid	0.800	0.800	0.800	0.276	0.276	0.276	0.042	0.042	0.276
PFHxS	Perfluorohexanesulphonic Acid	0.500	0.500	0.500	0.293	0.293	0.293	0.030	0.030	0.293
PFNA	Perfluorononanoic Acid	0.500	0.500	0.500	0.279	0.279	0.279	0.044	0.044	0.279
PFOA	Perfluorooctanoic Acid	0.500	0.500	0.500	0.420	0.420	0.420	0.135	0.135	0.420
PFOS	Perfluorooctanesulfonic Acid	0.500	0.500	0.500	0.600	0.600	0.600	1.046	1.046	0.600
PFOSA	Perfluorooctanesulfonamide	0.500	0.500	0.500	0.287	0.287	0.287	0.005	0.005	0.287
PFTeA	Perfluorotetradecanoic Acid	0.500	0.500	0.500	0.005	0.005	0.005	0.005	0.005	0.005
PFTriA	Perfluorotridecanoic Acid	0.400	0.400	0.400	0.005	0.005	0.005	0.005	0.005	0.005
PFUnDA	Perfluoroundecanoic Acid	0.800	0.800	0.800	0.235	0.235	0.235	0.028	0.028	0.235

[1] Data and LODs reported in Houde, M.; Wells, R. S.; Fair, P. A.; Bossart, G. D.; Hohn, A. A.; Rowles, T. K.; Sweeney, J. C.; Solomon, K. R.; Muir, D. C. Polyfluoroalkyl compounds in free-ranging bottlenose dolphins (Tursiops truncatus) from the Gulf of Mexico and the Atlantic Ocean. *Environ Sci Technol* **2005**, *39* (17), 6591-6598. DOI: 10.1021/es0506556 From NLM Medline.

Table S3. PFAS tertile sample sizes, means (ng/g), and cutoff ranges.

PFAS	Measure	Tertile 1	Tertile 2	Tertile 3
PFDA	n	93	91	92
	Median	5.06	19.70	129.09
	Range	2.06 – 11.07	>11.07 – 63.40	>63.40
PFDS	n	41	41	41
	Median	1.15	2.26	16.98
	Range	0.76 – 1.44	>1.44 – 4.11	>4.11
PFDoDA	n	92	92	92
	Median	0.77	2.44	9.55
	Range	0.18 – 1.45	>1.45 – 4.73	>4.73
PFHpA	n	59	21	40
	Median	0.76	1.00	5.07
	Range	0.12 – 0.83	>0.83 – 1.12	>1.12
PFHxS	n	92	93	91
	Median	8.02	21.80	111.40
	Range	2.31 – 13.30	>13.30 – 44.41	>44.41
PFNA	n	92	93	91
	Median	6.23	15.89	72.30
	Min-Max	0.25 – 9.42	>9.42 – 33.78	>33.78
PFOA	n	92	92	92
	Median	3.67	10.54	42.65
	Min-Max	0.25 – 5.78	>5.78 – 23.24	>23.24
PFOS	n	80	79	79
	Median	250.87	706.06	1751.26
	Min-Max	69.21 – 409.00	>409.00 – 1055.24	>1055.24
PFOSA	n	80	80	80
	Median	0.50	4.39	29.92
	Min-Max	0.21 – 0.96	>0.96 – 14.20	>14.20
PFTeA	n	71	21	46
	Median	0.40	0.61	1.74
	Min-Max	0.21 – 0.40	>0.40 – 0.84	>0.84
PFTriA	n	39	33	36
	Median	0.40	1.61	4.14
	Min-Max	0.40	>0.40 – 2.20	>2.20
PFUnDA	n	92	93	91
	Median	5.87	12.80	64.14
	Min-Max	1.78 – 8.22	>8.22 – 32.01	>32.01

Table S4. Distributions for each PFAS-cortisol model.
Note: each respective PFAS-Cortisol model (e.g., stratified, unadjusted, etc.) was fit using the below distributions.

Model Distributions			
**PFAS**	**Cortisol**		
	**Free**	**Bound**	**Total**
**PFOS**	Weibull	Lognormal	Weibull
**PFOA**	Weibull	Lognormal	Weibull
**PFOSA**	Weibull	Lognormal	Weibull
**PFDA**	Weibull	Lognormal	Weibull
**PFDoDA**	Weibull	Lognormal	Weibull
**PFDS**	Lognormal	Lognormal	Weibull
**PFHxS**	Weibull	Lognormal	Weibull
**PFHpA**	Lognormal	Lognormal	Weibull
**PFNA**	Weibull	Lognormal	Weibull
**PFTeA**	Weibull	Lognormal	Weibull
**PFTriA**	Weibull	Lognormal	Weibull
**PFUnDA**	Weibull	Lognormal	Weibull
**ΣPFCA**	Weibull	Lognormal	Weibull
**ΣPFSA**	Weibull	Lognormal	Weibull
**ΣPFAS**	Weibull	Lognormal	Weibull

Table S5. Principal component analysis with log transformed PFAS. Both perfluoroalkyl carboxylic acid (CA) and perfluoroalkyl sulfonic acids and sulfonamides (SA) were included.

log(PFAS)	Class	Measure	PCA1	PCA2	PCA3
		Eigenvalue	8.63	0.89	0.64
		Proportion	78.5%	8.1%	5.8%
		Cumulative	78.5%	86.6%	92.4%
PFDA	CA	Loading	0.33	-0.19	0.12
PFDoDA	CA	Loading	0.32	0.27	-0.05
PFNA	CA	Loading	0.33	-0.19	0.08
PFOA	CA	Loading	0.32	-0.30	-0.02
PFHpA	CA	Loading	0.25	0.06	0.66
PFTeA	CA	Loading	0.25	0.67	-0.09
PFTriA	CA	Loading	0.31	0.35	0.12
PFUnDA	CA	Loading	0.32	-0.19	-0.05
PFDS	SA	Loading	0.32	0.14	-0.29
PFHxS	SA	Loading	0.26	-0.17	-0.64
PFOSA	SA	Loading	0.31	-0.31	0.16

Table S6. Regression coefficients, 95% confidence intervals, and sample sizes (n) for each adjusted model.

Stratification	Cortisol	PFAS Tertile	Quantile Ratio	95% Confidence Interval		n
None	Free	PFDA Tertile 2 vs. 1	1.110	0.873	1.412	211
None	Free	PFDA Tertile 3 vs. 1	1.295	0.937	1.790	
None	Free	PFDoDA Tertile 2 vs. 1	1.223	0.992	1.509	211
None	Free	PFDoDA Tertile 3 vs. 1	1.120	0.885	1.417	
None	Free	PFHpA Tertile 2 vs. 1	0.853	0.360	2.022	85
None	Free	PFHpA Tertile 3 vs. 1	0.933	0.587	1.485	
None	Free	PFNA Tertile 2 vs. 1	0.881	0.692	1.120	211
None	Free	PFNA Tertile 3 vs. 1	0.873	0.613	1.243	
None	Free	PFOA Tertile 2 vs. 1	0.682	0.546	0.850	211
None	Free	PFOA Tertile 3 vs. 1	0.765	0.562	1.041	
None	Free	PFTeA Tertile 2 vs. 1	1.276	0.761	2.139	102
None	Free	PFTeA Tertile 3 vs. 1	0.852	0.688	1.055	
None	Free	PFTriA Tertile 2 vs. 1	1.502	1.082	2.085	77
None	Free	PFTriA Tertile 3 vs. 1	1.900	1.310	2.755	
None	Free	PFUnDA Tertile 2 vs. 1	0.945	0.734	1.217	211
None	Free	PFUnDA Tertile 3 vs. 1	0.918	0.668	1.263	
None	Free	PFDS Tertile 2 vs. 1	0.848	0.452	1.591	88
None	Free	PFDS Tertile 3 vs. 1	1.272	0.538	3.009	
None	Free	PFHxS Tertile 2 vs. 1	0.876	0.700	1.096	211
None	Free	PFHxS Tertile 3 vs. 1	0.777	0.605	0.997	
None	Free	PFOS Tertile 2 vs. 1	0.849	0.654	1.103	192
None	Free	PFOS Tertile 2 vs. 2	0.743	0.570	0.968	
None	Free	PFOSA Tertile 2 vs. 1	0.789	0.604	1.031	184
None	Free	PFOSA Tertile 3 vs. 1	0.929	0.651	1.324	
None	Free	ΣPFCA Tertile 2 vs. 1	0.691	0.491	0.973	150
None	Free	ΣPFCA Tertile 3 vs. 1	0.678	0.433	1.060	
None	Free	ΣPFSA Tertile 2 vs. 1	0.807	0.618	1.053	150
None	Free	ΣPFSA Tertile 3 vs. 1	0.650	0.504	0.839	
None	Free	ΣPFAS Tertile 2 vs. 1	0.885	0.674	1.163	192
None	Free	ΣPFAS Tertile 3 vs. 1	0.794	0.597	1.058	

None	Bound	PFDA Tertile 2 vs. 1	1.265	1.096	1.460	219
None	Bound	PFDA Tertile 3 vs. 1	1.286	1.005	1.647	
None	Bound	PFDoDA Tertile 2 vs. 1	1.297	1.149	1.464	219
None	Bound	PFDoDA Tertile 3 vs. 1	1.227	1.037	1.451	
None	Bound	PFHpA Tertile 2 vs. 1	1.061	0.778	1.445	89
None	Bound	PFHpA Tertile 3 vs. 1	1.071	0.848	1.352	
None	Bound	PFNA Tertile 2 vs. 1	1.092	0.947	1.260	219
None	Bound	PFNA Tertile 3 vs. 1	1.045	0.823	1.328	
None	Bound	PFOA Tertile 2 vs. 1	1.009	0.873	1.166	219
None	Bound	PFOA Tertile 3 vs. 1	0.905	0.745	1.099	
None	Bound	PFTeA Tertile 2 vs. 1	1.537	1.130	2.090	104
None	Bound	PFTeA Tertile 3 vs. 1	1.356	1.135	1.620	
None	Bound	PFTriA Tertile 2 vs. 1	0.949	0.708	1.270	77
None	Bound	PFTriA Tertile 3 vs. 1	1.091	0.757	1.573	
None	Bound	PFUnDA Tertile 2 vs. 1	1.225	1.059	1.417	219
None	Bound	PFUnDA Tertile 3 vs. 1	1.290	1.038	1.604	
None	Bound	PFDS Tertile 2 vs. 1	1.246	1.006	1.544	92
None	Bound	PFDS Tertile 3 vs. 1	1.613	1.199	2.170	
None	Bound	PFHxS Tertile 2 vs. 1	0.931	0.795	1.091	219
None	Bound	PFHxS Tertile 3 vs. 1	1.011	0.855	1.196	
None	Bound	PFOS Tertile 2 vs. 1	1.161	0.990	1.360	200
None	Bound	PFOS Tertile 2 vs. 2	1.166	0.981	1.386	
None	Bound	PFOSA Tertile 2 vs. 1	1.093	0.927	1.289	191
None	Bound	PFOSA Tertile 3 vs. 1	0.900	0.635	1.276	
None	Bound	ΣPFCA Tertile 2 vs. 1	1.074	0.892	1.293	156
None	Bound	ΣPFCA Tertile 3 vs. 1	1.059	0.780	1.439	
None	Bound	ΣPFSA Tertile 2 vs. 1	1.241	1.036	1.487	156
None	Bound	ΣPFSA Tertile 3 vs. 1	1.233	1.022	1.487	
None	Bound	ΣPFAS Tertile 2 vs. 1	1.194	1.020	1.398	200
None	Bound	ΣPFAS Tertile 3 vs. 1	1.209	1.015	1.440	
None	Total	PFDA Tertile 2 vs. 1	1.169	0.988	1.383	211
None	Total	PFDA Tertile 3 vs. 1	1.289	1.021	1.627	

None	Total	PFDoDA Tertile 2 vs. 1	1.243	1.074	1.439	211
None	Total	PFDoDA Tertile 3 vs. 1	1.211	1.034	1.418	
None	Total	PFHpA Tertile 2 vs. 1	1.196	0.836	1.712	85
None	Total	PFHpA Tertile 3 vs. 1	0.980	0.820	1.172	
None	Total	PFNA Tertile 2 vs. 1	0.937	0.795	1.104	211
None	Total	PFNA Tertile 3 vs. 1	0.935	0.756	1.158	
None	Total	PFOA Tertile 2 vs. 1	0.795	0.680	0.930	211
None	Total	PFOA Tertile 3 vs. 1	0.808	0.659	0.991	
None	Total	PFTeA Tertile 2 vs. 1	1.502	1.070	2.110	102
None	Total	PFTeA Tertile 3 vs. 1	0.992	0.856	1.151	
None	Total	PFTriA Tertile 2 vs. 1	1.256	0.995	1.584	77
None	Total	PFTriA Tertile 3 vs. 1	1.595	1.237	2.055	
None	Total	PFUnDA Tertile 2 vs. 1	0.998	0.833	1.195	211
None	Total	PFUnDA Tertile 3 vs. 1	1.001	0.793	1.262	
None	Total	PFDS Tertile 2 vs. 1	1.083	0.851	1.380	88
None	Total	PFDS Tertile 3 vs. 1	1.315	0.930	1.860	
None	Total	PFHxS Tertile 2 vs. 1	0.888	0.766	1.030	211
None	Total	PFHxS Tertile 3 vs. 1	0.845	0.713	1.001	
None	Total	PFOS Tertile 2 vs. 1	0.951	0.806	1.123	192
None	Total	PFOS Tertile 2 vs. 2	0.867	0.717	1.048	
None	Total	PFOSA Tertile 2 vs. 1	0.883	0.732	1.066	184
None	Total	PFOSA Tertile 3 vs. 1	0.943	0.729	1.220	
None	Total	ΣPFCA Tertile 2 vs. 1	0.797	0.634	1.003	150
None	Total	ΣPFCA Tertile 3 vs. 1	0.775	0.579	1.036	
None	Total	ΣPFSA Tertile 2 vs. 1	0.923	0.774	1.100	150
None	Total	ΣPFSA Tertile 3 vs. 1	0.802	0.664	0.969	
None	Total	ΣPFAS Tertile 2 vs. 1	0.988	0.829	1.179	192
None	Total	ΣPFAS Tertile 3 vs. 1	0.930	0.760	1.138	
Female	Free	PFDA Tertile 2 vs. 1	0.713	0.468	1.087	61
Female	Free	PFDA Tertile 3 vs. 1	0.691	0.432	1.106	
Female	Free	PFDoDA Tertile 2 vs. 1	0.882	0.555	1.402	61
Female	Free	PFDoDA Tertile 3 vs. 1	0.866	0.548	1.367	

Female	Free	PFHpA Tertile 2 vs. 1	1.325	0.943	1.862	18
Female	Free	PFHpA Tertile 3 vs. 1	0.708	0.416	1.203	
Female	Free	PFNA Tertile 2 vs. 1	0.838	0.523	1.345	61
Female	Free	PFNA Tertile 3 vs. 1	0.788	0.392	1.585	
Female	Free	PFOA Tertile 2 vs. 1	0.544	0.387	0.765	61
Female	Free	PFOA Tertile 3 vs. 1	0.591	0.388	0.900	
Female	Free	PFTeA Tertile 2 vs. 1	3.239	1.507	6.963	41
Female	Free	PFTeA Tertile 3 vs. 1	1.085	0.693	1.699	
Female	Free	PFTriA Tertile 2 vs. 1	1.424	0.894	2.268	31
Female	Free	PFTriA Tertile 3 vs. 1	1.592	0.884	2.869	
Female	Free	PFUnDA Tertile 2 vs. 1	1.144	0.643	2.033	61
Female	Free	PFUnDA Tertile 3 vs. 1	1.283	0.645	2.552	
Female	Free	PFDS Tertile 2 vs. 1	1.346	1.099	1.650	18
Female	Free	PFDS Tertile 3 vs. 1	0.948	0.502	1.788	
Female	Free	PFHxS Tertile 2 vs. 1	1.268	0.892	1.801	61
Female	Free	PFHxS Tertile 3 vs. 1	0.835	0.583	1.196	
Female	Free	PFOS Tertile 2 vs. 1	1.005	0.624	1.618	55
Female	Free	PFOS Tertile 2 vs. 2	0.603	0.369	0.986	
Female	Free	PFOSA Tertile 2 vs. 1	0.723	0.445	1.173	61
Female	Free	PFOSA Tertile 3 vs. 1	1.019	0.445	2.333	
Female	Free	ΣPFCA Tertile 2 vs. 1	0.691	0.491	0.973	53
Female	Free	ΣPFCA Tertile 3 vs. 1	0.678	0.433	1.060	
Female	Free	ΣPFSA Tertile 2 vs. 1	0.807	0.618	1.053	53
Female	Free	ΣPFSA Tertile 3 vs. 1	0.650	0.504	0.839	
Female	Free	ΣPFAS Tertile 2 vs. 1	0.885	0.674	1.163	55
Female	Free	ΣPFAS Tertile 3 vs. 1	0.794	0.597	1.058	
Female	Bound	PFDA Tertile 2 vs. 1	1.274	0.952	1.706	61
Female	Bound	PFDA Tertile 3 vs. 1	0.987	0.631	1.545	
Female	Bound	PFDoDA Tertile 2 vs. 1	1.116	0.872	1.427	61
Female	Bound	PFDoDA Tertile 3 vs. 1	1.112	0.837	1.477	
Female	Bound	PFHpA Tertile 2 vs. 1	1.542	1.024	2.321	18
Female	Bound	PFHpA Tertile 3 vs. 1	1.403	0.830	2.374	

Female	Bound	PFNA Tertile 2 vs. 1	1.014	0.748	1.374	61
Female	Bound	PFNA Tertile 3 vs. 1	1.362	0.873	2.123	
Female	Bound	PFOA Tertile 2 vs. 1	1.187	0.908	1.551	61
Female	Bound	PFOA Tertile 3 vs. 1	1.323	0.964	1.817	
Female	Bound	PFTeA Tertile 2 vs. 1	1.437	0.551	3.749	41
Female	Bound	PFTeA Tertile 3 vs. 1	1.312	0.946	1.820	
Female	Bound	PFTriA Tertile 2 vs. 1	0.799	0.565	1.130	31
Female	Bound	PFTriA Tertile 3 vs. 1	0.887	0.530	1.483	
Female	Bound	PFUnDA Tertile 2 vs. 1	1.574	1.059	2.341	61
Female	Bound	PFUnDA Tertile 3 vs. 1	1.516	0.968	2.373	
Female	Bound	PFDS Tertile 2 vs. 1	1.869	1.295	2.699	18
Female	Bound	PFDS Tertile 3 vs. 1	0.841	0.426	1.657	
Female	Bound	PFHxS Tertile 2 vs. 1	1.077	0.781	1.484	61
Female	Bound	PFHxS Tertile 3 vs. 1	1.301	0.958	1.768	
Female	Bound	PFOS Tertile 2 vs. 1	1.335	0.938	1.899	55
Female	Bound	PFOS Tertile 2 vs. 2	1.410	0.998	1.991	
Female	Bound	PFOSA Tertile 2 vs. 1	0.951	0.698	1.295	61
Female	Bound	PFOSA Tertile 3 vs. 1	0.767	0.420	1.399	
Female	Bound	ΣPFCA Tertile 2 vs. 1	1.074	0.892	1.293	53
Female	Bound	ΣPFCA Tertile 3 vs. 1	1.059	0.780	1.439	
Female	Bound	ΣPFSA Tertile 2 vs. 1	1.241	1.036	1.487	53
Female	Bound	ΣPFSA Tertile 3 vs. 1	1.233	1.022	1.487	
Female	Bound	ΣPFAS Tertile 2 vs. 1	1.194	1.020	1.398	55
Female	Bound	ΣPFAS Tertile 3 vs. 1	1.209	1.015	1.440	
Female	Total	PFDA Tertile 2 vs. 1	0.847	0.635	1.129	61
Female	Total	PFDA Tertile 3 vs. 1	0.749	0.530	1.057	
Female	Total	PFDoDA Tertile 2 vs. 1	0.920	0.688	1.229	61
Female	Total	PFDoDA Tertile 3 vs. 1	0.936	0.690	1.271	
Female	Total	PFHpA Tertile 2 vs. 1	1.060	0.703	1.599	18
Female	Total	PFHpA Tertile 3 vs. 1	0.877	0.570	1.348	
Female	Total	PFNA Tertile 2 vs. 1	0.915	0.659	1.271	61
Female	Total	PFNA Tertile 3 vs. 1	0.991	0.606	1.619	

Female	Total	PFOA Tertile 2 vs. 1	0.684	0.547	0.856	61
Female	Total	PFOA Tertile 3 vs. 1	0.757	0.572	1.002	
Female	Total	PFTeA Tertile 2 vs. 1	2.440	1.379	4.318	41
Female	Total	PFTeA Tertile 3 vs. 1	1.076	0.799	1.450	
Female	Total	PFTriA Tertile 2 vs. 1	1.166	0.862	1.576	31
Female	Total	PFTriA Tertile 3 vs. 1	1.423	1.009	2.005	
Female	Total	PFUnDA Tertile 2 vs. 1	1.178	0.703	1.975	61
Female	Total	PFUnDA Tertile 3 vs. 1	1.272	0.701	2.307	
Female	Total	PFDS Tertile 2 vs. 1	1.706	1.405	2.071	18
Female	Total	PFDS Tertile 3 vs. 1	0.846	0.567	1.261	
Female	Total	PFHxS Tertile 2 vs. 1	1.237	0.976	1.568	61
Female	Total	PFHxS Tertile 3 vs. 1	0.962	0.780	1.187	
Female	Total	PFOS Tertile 2 vs. 1	1.112	0.822	1.503	55
Female	Total	PFOS Tertile 2 vs. 2	0.791	0.580	1.079	
Female	Total	PFOSA Tertile 2 vs. 1	0.862	0.629	1.180	61
Female	Total	PFOSA Tertile 3 vs. 1	0.982	0.598	1.614	
Female	Total	ΣPFCA Tertile 2 vs. 1	0.797	0.634	1.003	53
Female	Total	ΣPFCA Tertile 3 vs. 1	0.775	0.579	1.036	
Female	Total	ΣPFSA Tertile 2 vs. 1	0.923	0.774	1.100	53
Female	Total	ΣPFSA Tertile 3 vs. 1	0.802	0.664	0.969	
Female	Total	ΣPFAS Tertile 2 vs. 1	0.988	0.829	1.179	55
Female	Total	ΣPFAS Tertile 3 vs. 1	0.930	0.760	1.138	
Male	Free	PFDA Tertile 2 vs. 1	1.261	0.954	1.666	150
Male	Free	PFDA Tertile 3 vs. 1	1.568	1.058	2.323	
Male	Free	PFDoDA Tertile 2 vs. 1	1.258	0.980	1.615	150
Male	Free	PFDoDA Tertile 3 vs. 1	1.107	0.827	1.481	
Male	Free	PFHpA Tertile 2 vs. 1	0.707	0.233	2.145	67
Male	Free	PFHpA Tertile 3 vs. 1	0.847	0.478	1.500	
Male	Free	PFNA Tertile 2 vs. 1	0.818	0.624	1.071	150
Male	Free	PFNA Tertile 3 vs. 1	0.833	0.539	1.286	
Male	Free	PFOA Tertile 2 vs. 1	0.703	0.543	0.911	150
Male	Free	PFOA Tertile 3 vs. 1	0.825	0.568	1.198	

Male	Free	PFTeA Tertile 2 vs. 1	1.040	0.555	1.948	61
Male	Free	PFTeA Tertile 3 vs. 1	0.774	0.608	0.986	
Male	Free	PFTriA Tertile 2 vs. 1	1.632	1.084	2.457	46
Male	Free	PFTriA Tertile 3 vs. 1	2.380	1.495	3.787	
Male	Free	PFUnDA Tertile 2 vs. 1	0.935	0.705	1.239	150
Male	Free	PFUnDA Tertile 3 vs. 1	0.846	0.575	1.243	
Male	Free	PFDS Tertile 2 vs. 1	0.720	0.360	1.440	70
Male	Free	PFDS Tertile 3 vs. 1	1.152	0.448	2.962	
Male	Free	PFHxS Tertile 2 vs. 1	0.825	0.628	1.084	150
Male	Free	PFHxS Tertile 3 vs. 1	0.762	0.563	1.032	
Male	Free	PFOS Tertile 2 vs. 1	0.789	0.579	1.075	137
Male	Free	PFOS Tertile 2 vs. 2	0.770	0.575	1.031	
Male	Free	PFOSA Tertile 2 vs. 1	0.856	0.622	1.178	123
Male	Free	PFOSA Tertile 3 vs. 1	0.946	0.636	1.408	
Male	Free	ΣPFCA Tertile 2 vs. 1	0.691	0.491	0.973	97
Male	Free	ΣPFCA Tertile 3 vs. 1	0.678	0.433	1.060	
Male	Free	ΣPFSA Tertile 2 vs. 1	0.807	0.618	1.053	97
Male	Free	ΣPFSA Tertile 3 vs. 1	0.650	0.504	0.839	
Male	Free	ΣPFAS Tertile 2 vs. 1	0.885	0.674	1.163	137
Male	Free	ΣPFAS Tertile 3 vs. 1	0.794	0.597	1.058	
Male	Bound	PFDA Tertile 2 vs. 1	1.222	1.043	1.433	158
Male	Bound	PFDA Tertile 3 vs. 1	1.409	1.064	1.866	
Male	Bound	PFDoDA Tertile 2 vs. 1	1.321	1.149	1.520	158
Male	Bound	PFDoDA Tertile 3 vs. 1	1.210	0.995	1.471	
Male	Bound	PFHpA Tertile 2 vs. 1	0.977	0.666	1.434	71
Male	Bound	PFHpA Tertile 3 vs. 1	1.003	0.763	1.319	
Male	Bound	PFNA Tertile 2 vs. 1	1.088	0.921	1.285	158
Male	Bound	PFNA Tertile 3 vs. 1	0.894	0.657	1.215	
Male	Bound	PFOA Tertile 2 vs. 1	0.939	0.800	1.103	158
Male	Bound	PFOA Tertile 3 vs. 1	0.767	0.611	0.961	
Male	Bound	PFTeA Tertile 2 vs. 1	1.489	1.072	2.067	63
Male	Bound	PFTeA Tertile 3 vs. 1	1.433	1.167	1.761	

Male	Bound	PFTriA Tertile 2 vs. 1	1.148	0.735	1.793	46
Male	Bound	PFTriA Tertile 3 vs. 1	1.111	0.657	1.881	
Male	Bound	PFUnDA Tertile 2 vs. 1	1.106	0.944	1.297	158
Male	Bound	PFUnDA Tertile 3 vs. 1	1.241	0.952	1.618	
Male	Bound	PFDS Tertile 2 vs. 1	1.191	0.942	1.507	74
Male	Bound	PFDS Tertile 3 vs. 1	1.747	1.282	2.380	
Male	Bound	PFHxS Tertile 2 vs. 1	0.897	0.751	1.072	158
Male	Bound	PFHxS Tertile 3 vs. 1	0.893	0.728	1.094	
Male	Bound	PFOS Tertile 2 vs. 1	1.124	0.945	1.336	145
Male	Bound	PFOS Tertile 2 vs. 2	1.034	0.839	1.273	
Male	Bound	PFOSA Tertile 2 vs. 1	1.120	0.926	1.355	130
Male	Bound	PFOSA Tertile 3 vs. 1	0.980	0.664	1.444	
Male	Bound	ΣPFCA Tertile 2 vs. 1	1.074	0.892	1.293	103
Male	Bound	ΣPFCA Tertile 3 vs. 1	1.059	0.780	1.439	
Male	Bound	ΣPFSA Tertile 2 vs. 1	1.241	1.036	1.487	103
Male	Bound	ΣPFSA Tertile 3 vs. 1	1.233	1.022	1.487	
Male	Bound	ΣPFAS Tertile 2 vs. 1	1.194	1.020	1.398	145
Male	Bound	ΣPFAS Tertile 3 vs. 1	1.209	1.015	1.440	
Male	Total	PFDA Tertile 2 vs. 1	1.260	1.040	1.526	150
Male	Total	PFDA Tertile 3 vs. 1	1.562	1.191	2.048	
Male	Total	PFDoDA Tertile 2 vs. 1	1.290	1.084	1.537	150
Male	Total	PFDoDA Tertile 3 vs. 1	1.219	1.015	1.463	
Male	Total	PFHpA Tertile 2 vs. 1	1.173	0.709	1.940	67
Male	Total	PFHpA Tertile 3 vs. 1	0.930	0.746	1.160	
Male	Total	PFNA Tertile 2 vs. 1	0.869	0.723	1.045	150
Male	Total	PFNA Tertile 3 vs. 1	0.836	0.657	1.065	
Male	Total	PFOA Tertile 2 vs. 1	0.797	0.666	0.953	150
Male	Total	PFOA Tertile 3 vs. 1	0.820	0.641	1.049	
Male	Total	PFTeA Tertile 2 vs. 1	1.238	0.825	1.857	61
Male	Total	PFTeA Tertile 3 vs. 1	0.964	0.804	1.155	
Male	Total	PFTriA Tertile 2 vs. 1	1.335	0.970	1.838	46
Male	Total	PFTriA Tertile 3 vs. 1	1.731	1.215	2.467	

Male	Total	PFUnDA Tertile 2 vs. 1	0.973	0.802	1.181	150
Male	Total	PFUnDA Tertile 3 vs. 1	0.973	0.739	1.281	
Male	Total	PFDS Tertile 2 vs. 1	0.994	0.760	1.299	70
Male	Total	PFDS Tertile 3 vs. 1	1.341	0.928	1.937	
Male	Total	PFHxS Tertile 2 vs. 1	0.844	0.709	1.005	150
Male	Total	PFHxS Tertile 3 vs. 1	0.810	0.660	0.994	
Male	Total	PFOS Tertile 2 vs. 1	0.905	0.752	1.090	137
Male	Total	PFOS Tertile 2 vs. 2	0.861	0.696	1.065	
Male	Total	PFOSA Tertile 2 vs. 1	0.933	0.751	1.158	123
Male	Total	PFOSA Tertile 3 vs. 1	0.992	0.746	1.319	
Male	Total	ΣPFCA Tertile 2 vs. 1	0.797	0.634	1.003	97
Male	Total	ΣPFCA Tertile 3 vs. 1	0.775	0.579	1.036	
Male	Total	ΣPFSA Tertile 2 vs. 1	0.923	0.774	1.100	97
Male	Total	ΣPFSA Tertile 3 vs. 1	0.802	0.664	0.969	
Male	Total	ΣPFAS Tertile 2 vs. 1	0.988	0.829	1.179	137
Male	Total	ΣPFAS Tertile 3 vs. 1	0.930	0.760	1.138	
Juvenile	Free	PFDA Tertile 2 vs. 1	0.801	0.464	1.381	59
Juvenile	Free	PFDA Tertile 3 vs. 1	0.975	0.607	1.569	
Juvenile	Free	PFDoDA Tertile 2 vs. 1	0.766	0.465	1.263	59
Juvenile	Free	PFDoDA Tertile 3 vs. 1	0.746	0.462	1.204	
Juvenile	Free	PFHpA Tertile 2 vs. 1	0.216	0.067	0.701	20
Juvenile	Free	PFHpA Tertile 3 vs. 1	0.872	0.571	1.332	
Juvenile	Free	PFNA Tertile 2 vs. 1	0.819	0.486	1.379	59
Juvenile	Free	PFNA Tertile 3 vs. 1	0.656	0.330	1.305	
Juvenile	Free	PFOA Tertile 2 vs. 1	0.685	0.432	1.086	59
Juvenile	Free	PFOA Tertile 3 vs. 1	0.612	0.371	1.009	
Juvenile	Free	PFTeA Tertile 2 vs. 1	0.005	0.000	0.101	36
Juvenile	Free	PFTeA Tertile 3 vs. 1	0.506	0.301	0.851	
Juvenile	Free	PFTriA Tertile 2 vs. 1	1.119	0.590	2.123	26
Juvenile	Free	PFTriA Tertile 3 vs. 1	1.305	0.795	2.142	
Juvenile	Free	PFUnDA Tertile 2 vs. 1	0.678	0.407	1.129	59
Juvenile	Free	PFUnDA Tertile 3 vs. 1	0.723	0.439	1.189	

Juvenile	Free	PFDS Tertile 2 vs. 1	2.701	0.452	16.163	20
Juvenile	Free	PFDS Tertile 3 vs. 1	2.451	0.209	28.770	
Juvenile	Free	PFHxS Tertile 2 vs. 1	0.779	0.443	1.369	59
Juvenile	Free	PFHxS Tertile 3 vs. 1	0.511	0.331	0.790	
Juvenile	Free	PFOS Tertile 2 vs. 1	0.950	0.594	1.520	58
Juvenile	Free	PFOS Tertile 2 vs. 2	0.627	0.402	0.980	
Juvenile	Free	PFOSA Tertile 2 vs. 1	0.791	0.496	1.261	56
Juvenile	Free	PFOSA Tertile 3 vs. 1	4.397	0.446	43.359	
Juvenile	Free	ΣPFCA Tertile 2 vs. 1	0.691	0.491	0.973	51
Juvenile	Free	ΣPFCA Tertile 3 vs. 1	0.678	0.433	1.060	
Juvenile	Free	ΣPFSA Tertile 2 vs. 1	0.807	0.618	1.053	51
Juvenile	Free	ΣPFSA Tertile 3 vs. 1	0.650	0.504	0.839	
Juvenile	Free	ΣPFAS Tertile 2 vs. 1	0.885	0.674	1.163	58
Juvenile	Free	ΣPFAS Tertile 3 vs. 1	0.794	0.597	1.058	
Juvenile	Bound	PFDA Tertile 2 vs. 1	1.161	0.887	1.521	62
Juvenile	Bound	PFDA Tertile 3 vs. 1	1.093	0.795	1.502	
Juvenile	Bound	PFDoDA Tertile 2 vs. 1	1.025	0.828	1.268	62
Juvenile	Bound	PFDoDA Tertile 3 vs. 1	1.072	0.792	1.452	
Juvenile	Bound	PFHpA Tertile 2 vs. 1	0.837	0.602	1.165	22
Juvenile	Bound	PFHpA Tertile 3 vs. 1	1.151	0.879	1.506	
Juvenile	Bound	PFNA Tertile 2 vs. 1	1.219	0.866	1.715	62
Juvenile	Bound	PFNA Tertile 3 vs. 1	1.455	0.966	2.193	
Juvenile	Bound	PFOA Tertile 2 vs. 1	1.032	0.769	1.384	62
Juvenile	Bound	PFOA Tertile 3 vs. 1	0.993	0.706	1.398	
Juvenile	Bound	PFTeA Tertile 2 vs. 1	0.252	0.020	3.170	37
Juvenile	Bound	PFTeA Tertile 3 vs. 1	1.324	0.848	2.068	
Juvenile	Bound	PFTriA Tertile 2 vs. 1	0.922	0.620	1.370	26
Juvenile	Bound	PFTriA Tertile 3 vs. 1	0.985	0.638	1.520	
Juvenile	Bound	PFUnDA Tertile 2 vs. 1	1.317	1.023	1.695	62
Juvenile	Bound	PFUnDA Tertile 3 vs. 1	1.191	0.841	1.686	
Juvenile	Bound	PFDS Tertile 2 vs. 1	0.960	0.587	1.568	22
Juvenile	Bound	PFDS Tertile 3 vs. 1	1.098	0.535	2.251	

Juvenile	Bound	PFHxS Tertile 2 vs. 1	1.213	0.827	1.780	62
Juvenile	Bound	PFHxS Tertile 3 vs. 1	1.160	0.822	1.637	
Juvenile	Bound	PFOS Tertile 2 vs. 1	1.396	1.084	1.799	61
Juvenile	Bound	PFOS Tertile 2 vs. 2	1.229	0.942	1.603	
Juvenile	Bound	PFOSA Tertile 2 vs. 1	1.228	0.933	1.616	59
Juvenile	Bound	PFOSA Tertile 3 vs. 1	1.680	0.895	3.153	
Juvenile	Bound	ΣPFCA Tertile 2 vs. 1	1.074	0.892	1.293	53
Juvenile	Bound	ΣPFCA Tertile 3 vs. 1	1.059	0.780	1.439	
Juvenile	Bound	ΣPFSA Tertile 2 vs. 1	1.241	1.036	1.487	53
Juvenile	Bound	ΣPFSA Tertile 3 vs. 1	1.233	1.022	1.487	
Juvenile	Bound	ΣPFAS Tertile 2 vs. 1	1.194	1.020	1.398	61
Juvenile	Bound	ΣPFAS Tertile 3 vs. 1	1.209	1.015	1.440	
Juvenile	Total	PFDA Tertile 2 vs. 1	0.929	0.673	1.281	59
Juvenile	Total	PFDA Tertile 3 vs. 1	1.006	0.742	1.366	
Juvenile	Total	PFDoDA Tertile 2 vs. 1	0.868	0.634	1.189	59
Juvenile	Total	PFDoDA Tertile 3 vs. 1	0.892	0.680	1.171	
Juvenile	Total	PFHpA Tertile 2 vs. 1	0.458	0.363	0.579	20
Juvenile	Total	PFHpA Tertile 3 vs. 1	0.906	0.796	1.032	
Juvenile	Total	PFNA Tertile 2 vs. 1	0.908	0.659	1.252	59
Juvenile	Total	PFNA Tertile 3 vs. 1	0.834	0.573	1.214	
Juvenile	Total	PFOA Tertile 2 vs. 1	0.759	0.582	0.990	59
Juvenile	Total	PFOA Tertile 3 vs. 1	0.729	0.546	0.974	
Juvenile	Total	PFTeA Tertile 2 vs. 1	0.015	0.002	0.131	36
Juvenile	Total	PFTeA Tertile 3 vs. 1	0.694	0.502	0.960	
Juvenile	Total	PFTriA Tertile 2 vs. 1	1.121	0.726	1.731	26
Juvenile	Total	PFTriA Tertile 3 vs. 1	1.245	0.900	1.722	
Juvenile	Total	PFUnDA Tertile 2 vs. 1	0.858	0.636	1.159	59
Juvenile	Total	PFUnDA Tertile 3 vs. 1	0.868	0.654	1.152	
Juvenile	Total	PFDS Tertile 2 vs. 1	0.760	0.459	1.258	20
Juvenile	Total	PFDS Tertile 3 vs. 1	0.670	0.332	1.352	
Juvenile	Total	PFHxS Tertile 2 vs. 1	0.869	0.674	1.119	59
Juvenile	Total	PFHxS Tertile 3 vs. 1	0.647	0.511	0.818	

Juvenile	Total	PFOS Tertile 2 vs. 1	1.081	0.826	1.415	58
Juvenile	Total	PFOS Tertile 2 vs. 2	0.767	0.584	1.007	
Juvenile	Total	PFOSA Tertile 2 vs. 1	0.918	0.701	1.202	56
Juvenile	Total	PFOSA Tertile 3 vs. 1	3.245	0.886	11.883	
Juvenile	Total	ΣPFCA Tertile 2 vs. 1	0.797	0.634	1.003	51
Juvenile	Total	ΣPFCA Tertile 3 vs. 1	0.775	0.579	1.036	
Juvenile	Total	ΣPFSA Tertile 2 vs. 1	0.923	0.774	1.100	51
Juvenile	Total	ΣPFSA Tertile 3 vs. 1	0.802	0.664	0.969	
Juvenile	Total	ΣPFAS Tertile 2 vs. 1	0.988	0.829	1.179	58
Juvenile	Total	ΣPFAS Tertile 3 vs. 1	0.930	0.760	1.138	
Adult	Free	PFDA Tertile 2 vs. 1	1.237	0.981	1.561	186
Adult	Free	PFDA Tertile 3 vs. 1	1.272	0.918	1.764	
Adult	Free	PFDoDA Tertile 2 vs. 1	1.158	0.943	1.421	186
Adult	Free	PFDoDA Tertile 3 vs. 1	1.010	0.797	1.280	
Adult	Free	PFHpA Tertile 2 vs. 1	1.888	0.975	3.655	78
Adult	Free	PFHpA Tertile 3 vs. 1	0.813	0.450	1.469	
Adult	Free	PFNA Tertile 2 vs. 1	0.987	0.779	1.250	186
Adult	Free	PFNA Tertile 3 vs. 1	0.893	0.638	1.251	
Adult	Free	PFOA Tertile 2 vs. 1	0.741	0.597	0.919	186
Adult	Free	PFOA Tertile 3 vs. 1	0.726	0.546	0.965	
Adult	Free	PFTeA Tertile 2 vs. 1	1.545	0.968	2.467	81
Adult	Free	PFTeA Tertile 3 vs. 1	1.034	0.809	1.322	
Adult	Free	PFTriA Tertile 2 vs. 1	1.421	1.011	1.999	64
Adult	Free	PFTriA Tertile 3 vs. 1	1.843	1.314	2.587	
Adult	Free	PFUnDA Tertile 2 vs. 1	0.988	0.771	1.268	186
Adult	Free	PFUnDA Tertile 3 vs. 1	0.893	0.637	1.252	
Adult	Free	PFDS Tertile 2 vs. 1	0.508	0.250	1.033	81
Adult	Free	PFDS Tertile 3 vs. 1	0.655	0.282	1.520	
Adult	Free	PFHxS Tertile 2 vs. 1	0.967	0.781	1.197	186
Adult	Free	PFHxS Tertile 3 vs. 1	0.885	0.717	1.092	
Adult	Free	PFOS Tertile 2 vs. 1	0.903	0.693	1.177	164
Adult	Free	PFOS Tertile 2 vs. 2	0.835	0.631	1.105	

Adult	Free	PFOSA Tertile 2 vs. 1	0.796	0.592	1.070	156
Adult	Free	PFOSA Tertile 3 vs. 1	0.792	0.554	1.134	
Adult	Free	ΣPFCA Tertile 2 vs. 1	0.691	0.491	0.973	122
Adult	Free	ΣPFCA Tertile 3 vs. 1	0.678	0.433	1.060	
Adult	Free	ΣPFSA Tertile 2 vs. 1	0.807	0.618	1.053	122
Adult	Free	ΣPFSA Tertile 3 vs. 1	0.650	0.504	0.839	
Adult	Free	ΣPFAS Tertile 2 vs. 1	0.885	0.674	1.163	164
Adult	Free	ΣPFAS Tertile 3 vs. 1	0.794	0.597	1.058	
Adult	Bound	PFDA Tertile 2 vs. 1	1.321	1.086	1.607	192
Adult	Bound	PFDA Tertile 3 vs. 1	1.434	1.042	1.973	
Adult	Bound	PFDoDA Tertile 2 vs. 1	1.460	1.257	1.696	192
Adult	Bound	PFDoDA Tertile 3 vs. 1	1.384	1.141	1.679	
Adult	Bound	PFHpA Tertile 2 vs. 1	0.988	0.748	1.306	81
Adult	Bound	PFHpA Tertile 3 vs. 1	0.900	0.650	1.244	
Adult	Bound	PFNA Tertile 2 vs. 1	1.056	0.889	1.253	192
Adult	Bound	PFNA Tertile 3 vs. 1	1.046	0.789	1.388	
Adult	Bound	PFOA Tertile 2 vs. 1	1.012	0.850	1.206	192
Adult	Bound	PFOA Tertile 3 vs. 1	0.980	0.793	1.211	
Adult	Bound	PFTeA Tertile 2 vs. 1	1.243	0.908	1.702	83
Adult	Bound	PFTeA Tertile 3 vs. 1	1.059	0.839	1.337	
Adult	Bound	PFTriA Tertile 2 vs. 1	1.018	0.705	1.469	65
Adult	Bound	PFTriA Tertile 3 vs. 1	1.136	0.697	1.850	
Adult	Bound	PFUnDA Tertile 2 vs. 1	1.257	1.031	1.534	192
Adult	Bound	PFUnDA Tertile 3 vs. 1	1.382	1.031	1.853	
Adult	Bound	PFDS Tertile 2 vs. 1	1.239	0.974	1.577	84
Adult	Bound	PFDS Tertile 3 vs. 1	1.580	1.205	2.073	
Adult	Bound	PFHxS Tertile 2 vs. 1	0.921	0.786	1.080	192
Adult	Bound	PFHxS Tertile 3 vs. 1	1.087	0.907	1.303	
Adult	Bound	PFOS Tertile 2 vs. 1	1.102	0.899	1.350	169
Adult	Bound	PFOS Tertile 2 vs. 2	1.196	0.967	1.479	
Adult	Bound	PFOSA Tertile 2 vs. 1	1.140	0.920	1.412	161
Adult	Bound	PFOSA Tertile 3 vs. 1	1.013	0.702	1.463	

Adult	Bound	ΣPFCA Tertile 2 vs. 1	1.074	0.892	1.293	126
Adult	Bound	ΣPFCA Tertile 3 vs. 1	1.059	0.780	1.439	
Adult	Bound	ΣPFSA Tertile 2 vs. 1	1.241	1.036	1.487	126
Adult	Bound	ΣPFSA Tertile 3 vs. 1	1.233	1.022	1.487	
Adult	Bound	ΣPFAS Tertile 2 vs. 1	1.194	1.020	1.398	169
Adult	Bound	ΣPFAS Tertile 3 vs. 1	1.209	1.015	1.440	
Adult	Total	PFDA Tertile 2 vs. 1	1.270	1.075	1.501	186
Adult	Total	PFDA Tertile 3 vs. 1	1.320	1.045	1.667	
Adult	Total	PFDoDA Tertile 2 vs. 1	1.223	1.061	1.411	186
Adult	Total	PFDoDA Tertile 3 vs. 1	1.162	0.981	1.377	
Adult	Total	PFHpA Tertile 2 vs. 1	1.353	1.077	1.700	78
Adult	Total	PFHpA Tertile 3 vs. 1	0.924	0.731	1.168	
Adult	Total	PFNA Tertile 2 vs. 1	1.008	0.853	1.191	186
Adult	Total	PFNA Tertile 3 vs. 1	0.961	0.767	1.204	
Adult	Total	PFOA Tertile 2 vs. 1	0.853	0.729	1.000	186
Adult	Total	PFOA Tertile 3 vs. 1	0.795	0.652	0.970	
Adult	Total	PFTeA Tertile 2 vs. 1	1.635	1.214	2.202	81
Adult	Total	PFTeA Tertile 3 vs. 1	1.060	0.898	1.251	
Adult	Total	PFTriA Tertile 2 vs. 1	1.162	0.902	1.496	64
Adult	Total	PFTriA Tertile 3 vs. 1	1.479	1.107	1.976	
Adult	Total	PFUnDA Tertile 2 vs. 1	1.031	0.860	1.235	186
Adult	Total	PFUnDA Tertile 3 vs. 1	0.993	0.769	1.282	
Adult	Total	PFDS Tertile 2 vs. 1	0.950	0.743	1.215	81
Adult	Total	PFDS Tertile 3 vs. 1	1.162	0.771	1.751	
Adult	Total	PFHxS Tertile 2 vs. 1	0.941	0.816	1.085	186
Adult	Total	PFHxS Tertile 3 vs. 1	0.925	0.795	1.076	
Adult	Total	PFOS Tertile 2 vs. 1	0.992	0.838	1.174	164
Adult	Total	PFOS Tertile 2 vs. 2	0.950	0.775	1.164	
Adult	Total	PFOSA Tertile 2 vs. 1	0.898	0.727	1.110	156
Adult	Total	PFOSA Tertile 3 vs. 1	0.871	0.663	1.143	
Adult	Total	ΣPFCA Tertile 2 vs. 1	0.797	0.634	1.003	122
Adult	Total	ΣPFCA Tertile 3 vs. 1	0.775	0.579	1.036	

Adult	Total	ΣPFSA Tertile 2 vs. 1	0.923	0.774	1.100	122
Adult	Total	ΣPFSA Tertile 3 vs. 1	0.802	0.664	0.969	
Adult	Total	ΣPFAS Tertile 2 vs. 1	0.988	0.829	1.179	164
Adult	Total	ΣPFAS Tertile 3 vs. 1	0.930	0.760	1.138	

Table S7. Regression coefficients, 95% confidence intervals, and sample sizes (n) for each unadjusted model.

Stratification	Cortisol	PFAS Tertile	Quantile Ratio	95% Confidence Interval		n
None	Free	PFDA Tertile 2 vs. 1	1.291	1.032	1.615	247
None	Free	PFDA Tertile 3 vs. 1	1.038	0.850	1.268	
None	Free	PFDoDA Tertile 2 vs. 1	1.181	0.951	1.466	247
None	Free	PFDoDA Tertile 3 vs. 1	0.917	0.748	1.125	
None	Free	PFHpA Tertile 2 vs. 1	1.209	0.633	2.307	100
None	Free	PFHpA Tertile 3 vs. 1	0.775	0.488	1.233	
None	Free	PFNA Tertile 2 vs. 1	0.940	0.756	1.169	247
None	Free	PFNA Tertile 3 vs. 1	0.807	0.650	1.001	
None	Free	PFOA Tertile 2 vs. 1	0.664	0.546	0.807	247
None	Free	PFOA Tertile 3 vs. 1	0.738	0.604	0.902	
None	Free	PFTeA Tertile 2 vs. 1	0.532	0.372	0.760	119
None	Free	PFTeA Tertile 3 vs. 1	0.743	0.603	0.915	
None	Free	PFTriA Tertile 2 vs. 1	0.907	0.696	1.183	92
None	Free	PFTriA Tertile 3 vs. 1	0.837	0.582	1.203	
None	Free	PFUnDA Tertile 2 vs. 1	1.030	0.820	1.293	247
None	Free	PFUnDA Tertile 3 vs. 1	0.953	0.768	1.183	
None	Free	PFDS Tertile 2 vs. 1	0.716	0.402	1.274	103
None	Free	PFDS Tertile 3 vs. 1	1.371	0.869	2.164	
None	Free	PFHxS Tertile 2 vs. 1	0.826	0.665	1.027	247
None	Free	PFHxS Tertile 3 vs. 1	0.808	0.662	0.985	
None	Free	PFOS Tertile 2 vs. 1	0.905	0.729	1.125	222
None	Free	PFOS Tertile 2 vs. 2	0.744	0.614	0.901	
None	Free	PFOSA Tertile 2 vs. 1	0.714	0.566	0.900	214
None	Free	PFOSA Tertile 3 vs. 1	0.780	0.640	0.950	

None	Free	ΣPFCA Tertile 2 vs. 1	0.698	0.561	0.868	173
None	Free	ΣPFCA Tertile 3 vs. 1	0.668	0.552	0.808	
None	Free	ΣPFSA Tertile 2 vs. 1	0.821	0.657	1.026	173
None	Free	ΣPFSA Tertile 3 vs. 1	0.636	0.532	0.760	
None	Free	ΣPFAS Tertile 2 vs. 1	0.920	0.738	1.148	222
None	Free	ΣPFAS Tertile 3 vs. 1	0.789	0.648	0.961	
None	Bound	PFDA Tertile 2 vs. 1	1.030	0.894	1.187	257
None	Bound	PFDA Tertile 3 vs. 1	0.672	0.575	0.785	
None	Bound	PFDoDA Tertile 2 vs. 1	1.178	1.020	1.361	257
None	Bound	PFDoDA Tertile 3 vs. 1	0.786	0.671	0.921	
None	Bound	PFHpA Tertile 2 vs. 1	1.059	0.817	1.372	106
None	Bound	PFHpA Tertile 3 vs. 1	0.768	0.621	0.949	
None	Bound	PFNA Tertile 2 vs. 1	0.954	0.821	1.108	257
None	Bound	PFNA Tertile 3 vs. 1	0.677	0.581	0.790	
None	Bound	PFOA Tertile 2 vs. 1	0.899	0.770	1.049	257
None	Bound	PFOA Tertile 3 vs. 1	0.692	0.592	0.809	
None	Bound	PFTeA Tertile 2 vs. 1	1.032	0.800	1.332	123
None	Bound	PFTeA Tertile 3 vs. 1	1.070	0.873	1.311	
None	Bound	PFTriA Tertile 2 vs. 1	0.721	0.562	0.926	94
None	Bound	PFTriA Tertile 3 vs. 1	0.731	0.580	0.921	
None	Bound	PFUnDA Tertile 2 vs. 1	0.969	0.837	1.123	257
None	Bound	PFUnDA Tertile 3 vs. 1	0.698	0.596	0.818	
None	Bound	PFDS Tertile 2 vs. 1	0.967	0.776	1.205	109
None	Bound	PFDS Tertile 3 vs. 1	0.812	0.650	1.015	
None	Bound	PFHxS Tertile 2 vs. 1	0.961	0.814	1.133	257
None	Bound	PFHxS Tertile 3 vs. 1	0.958	0.811	1.130	
None	Bound	PFOS Tertile 2 vs. 1	0.891	0.749	1.059	230
None	Bound	PFOS Tertile 2 vs. 2	0.831	0.707	0.975	
None	Bound	PFOSA Tertile 2 vs. 1	0.933	0.800	1.089	223
None	Bound	PFOSA Tertile 3 vs. 1	0.617	0.521	0.731	
None	Bound	ΣPFCA Tertile 2 vs. 1	0.854	0.707	1.031	179
None	Bound	ΣPFCA Tertile 3 vs. 1	0.654	0.545	0.783	

None	Bound	ΣPFSA Tertile 2 vs. 1	1.000	0.819	1.221	179
None	Bound	ΣPFSA Tertile 3 vs. 1	0.907	0.748	1.099	
None	Bound	ΣPFAS Tertile 2 vs. 1	0.891	0.750	1.059	230
None	Bound	ΣPFAS Tertile 3 vs. 1	0.831	0.708	0.976	
None	Total	PFDA Tertile 2 vs. 1	1.254	1.076	1.462	247
None	Total	PFDA Tertile 3 vs. 1	0.932	0.822	1.058	
None	Total	PFDoDA Tertile 2 vs. 1	1.204	1.030	1.408	247
None	Total	PFDoDA Tertile 3 vs. 1	0.906	0.791	1.038	
None	Total	PFHpA Tertile 2 vs. 1	1.156	0.925	1.445	100
None	Total	PFHpA Tertile 3 vs. 1	0.810	0.641	1.023	
None	Total	PFNA Tertile 2 vs. 1	0.942	0.805	1.102	247
None	Total	PFNA Tertile 3 vs. 1	0.764	0.656	0.891	
None	Total	PFOA Tertile 2 vs. 1	0.755	0.652	0.874	247
None	Total	PFOA Tertile 3 vs. 1	0.736	0.635	0.853	
None	Total	PFTeA Tertile 2 vs. 1	0.692	0.520	0.920	119
None	Total	PFTeA Tertile 3 vs. 1	0.867	0.730	1.029	
None	Total	PFTriA Tertile 2 vs. 1	0.838	0.698	1.006	92
None	Total	PFTriA Tertile 3 vs. 1	0.846	0.655	1.091	
None	Total	PFUnDA Tertile 2 vs. 1	1.008	0.854	1.191	247
None	Total	PFUnDA Tertile 3 vs. 1	0.865	0.738	1.014	
None	Total	PFDS Tertile 2 vs. 1	0.985	0.777	1.248	103
None	Total	PFDS Tertile 3 vs. 1	1.114	0.902	1.375	
None	Total	PFHxS Tertile 2 vs. 1	0.860	0.734	1.008	247
None	Total	PFHxS Tertile 3 vs. 1	0.838	0.719	0.978	
None	Total	PFOS Tertile 2 vs. 1	0.921	0.787	1.078	222
None	Total	PFOS Tertile 2 vs. 2	0.768	0.659	0.896	
None	Total	PFOSA Tertile 2 vs. 1	0.802	0.680	0.947	214
None	Total	PFOSA Tertile 3 vs. 1	0.745	0.639	0.869	
None	Total	ΣPFCA Tertile 2 vs. 1	0.752	0.636	0.889	173
None	Total	ΣPFCA Tertile 3 vs. 1	0.666	0.578	0.768	
None	Total	ΣPFSA Tertile 2 vs. 1	0.868	0.731	1.031	173
None	Total	ΣPFSA Tertile 3 vs. 1	0.708	0.601	0.834	

None	Total	ΣPFAS Tertile 2 vs. 1	0.928	0.790	1.091	222
None	Total	ΣPFAS Tertile 3 vs. 1	0.798	0.684	0.931	
Female	Free	PFDA Tertile 2 vs. 1	0.942	0.645	1.375	81
Female	Free	PFDA Tertile 3 vs. 1	0.775	0.568	1.059	
Female	Free	PFDoDA Tertile 2 vs. 1	0.762	0.542	1.070	81
Female	Free	PFDoDA Tertile 3 vs. 1	0.687	0.503	0.938	
Female	Free	PFHpA Tertile 2 vs. 1	1.445	0.717	2.912	23
Female	Free	PFHpA Tertile 3 vs. 1	0.583	0.323	1.051	
Female	Free	PFNA Tertile 2 vs. 1	0.855	0.611	1.196	81
Female	Free	PFNA Tertile 3 vs. 1	0.726	0.527	1.000	
Female	Free	PFOA Tertile 2 vs. 1	0.579	0.434	0.774	81
Female	Free	PFOA Tertile 3 vs. 1	0.627	0.480	0.820	
Female	Free	PFTeA Tertile 2 vs. 1	0.630	0.398	0.998	52
Female	Free	PFTeA Tertile 3 vs. 1	0.788	0.572	1.086	
Female	Free	PFTriA Tertile 2 vs. 1	1.070	0.729	1.571	41
Female	Free	PFTriA Tertile 3 vs. 1	0.791	0.467	1.339	
Female	Free	PFUnDA Tertile 2 vs. 1	0.866	0.569	1.318	81
Female	Free	PFUnDA Tertile 3 vs. 1	0.819	0.539	1.243	
Female	Free	PFDS Tertile 2 vs. 1	0.586	0.286	1.201	23
Female	Free	PFDS Tertile 3 vs. 1	0.936	0.590	1.486	
Female	Free	PFHxS Tertile 2 vs. 1	1.240	0.857	1.794	81
Female	Free	PFHxS Tertile 3 vs. 1	0.810	0.628	1.045	
Female	Free	PFOS Tertile 2 vs. 1	1.054	0.759	1.463	72
Female	Free	PFOS Tertile 2 vs. 2	0.631	0.467	0.853	
Female	Free	PFOSA Tertile 2 vs. 1	0.643	0.443	0.933	80
Female	Free	PFOSA Tertile 3 vs. 1	0.703	0.533	0.926	
Female	Free	ΣPFCA Tertile 2 vs. 1	0.659	0.497	0.874	69
Female	Free	ΣPFCA Tertile 3 vs. 1	0.691	0.512	0.934	
Female	Free	ΣPFSA Tertile 2 vs. 1	1.110	0.817	1.508	69
Female	Free	ΣPFSA Tertile 3 vs. 1	0.646	0.500	0.835	
Female	Free	ΣPFAS Tertile 2 vs. 1	1.026	0.732	1.438	72
Female	Free	ΣPFAS Tertile 3 vs. 1	0.678	0.493	0.932	

Female	Bound	PFDA Tertile 2 vs. 1	1.207	0.824	1.767	81
Female	Bound	PFDA Tertile 3 vs. 1	0.622	0.425	0.911	
Female	Bound	PFDoDA Tertile 2 vs. 1	1.125	0.783	1.616	81
Female	Bound	PFDoDA Tertile 3 vs. 1	0.761	0.536	1.081	
Female	Bound	PFHpA Tertile 2 vs. 1	1.378	0.707	2.684	23
Female	Bound	PFHpA Tertile 3 vs. 1	0.946	0.579	1.543	
Female	Bound	PFNA Tertile 2 vs. 1	0.786	0.557	1.109	81
Female	Bound	PFNA Tertile 3 vs. 1	0.653	0.473	0.902	
Female	Bound	PFOA Tertile 2 vs. 1	0.921	0.648	1.308	81
Female	Bound	PFOA Tertile 3 vs. 1	0.760	0.547	1.057	
Female	Bound	PFTeA Tertile 2 vs. 1	1.134	0.775	1.658	52
Female	Bound	PFTeA Tertile 3 vs. 1	0.829	0.599	1.147	
Female	Bound	PFTriA Tertile 2 vs. 1	0.546	0.386	0.773	41
Female	Bound	PFTriA Tertile 3 vs. 1	0.688	0.473	1.001	
Female	Bound	PFUnDA Tertile 2 vs. 1	1.190	0.698	2.030	81
Female	Bound	PFUnDA Tertile 3 vs. 1	0.754	0.442	1.287	
Female	Bound	PFDS Tertile 2 vs. 1	1.223	0.760	1.968	23
Female	Bound	PFDS Tertile 3 vs. 1	0.771	0.488	1.219	
Female	Bound	PFHxS Tertile 2 vs. 1	0.975	0.696	1.364	81
Female	Bound	PFHxS Tertile 3 vs. 1	1.170	0.867	1.579	
Female	Bound	PFOS Tertile 2 vs. 1	0.818	0.525	1.275	72
Female	Bound	PFOS Tertile 2 vs. 2	0.920	0.604	1.401	
Female	Bound	PFOSA Tertile 2 vs. 1	1.045	0.747	1.462	80
Female	Bound	PFOSA Tertile 3 vs. 1	0.575	0.410	0.808	
Female	Bound	ΣPFCA Tertile 2 vs. 1	0.810	0.564	1.163	69
Female	Bound	ΣPFCA Tertile 3 vs. 1	0.602	0.435	0.832	
Female	Bound	ΣPFSA Tertile 2 vs. 1	1.172	0.809	1.697	69
Female	Bound	ΣPFSA Tertile 3 vs. 1	1.084	0.752	1.561	
Female	Bound	ΣPFAS Tertile 2 vs. 1	0.827	0.528	1.295	72
Female	Bound	ΣPFAS Tertile 3 vs. 1	0.911	0.599	1.384	
Female	Total	PFDA Tertile 2 vs. 1	1.014	0.787	1.306	81
Female	Total	PFDA Tertile 3 vs. 1	0.729	0.588	0.904	

Female	Total	PFDoDA Tertile 2 vs. 1	0.856	0.666	1.102	81
Female	Total	PFDoDA Tertile 3 vs. 1	0.708	0.565	0.888	
Female	Total	PFHpA Tertile 2 vs. 1	1.359	0.925	1.999	23
Female	Total	PFHpA Tertile 3 vs. 1	0.718	0.511	1.008	
Female	Total	PFNA Tertile 2 vs. 1	0.883	0.688	1.133	81
Female	Total	PFNA Tertile 3 vs. 1	0.715	0.577	0.886	
Female	Total	PFOA Tertile 2 vs. 1	0.683	0.543	0.860	81
Female	Total	PFOA Tertile 3 vs. 1	0.664	0.548	0.805	
Female	Total	PFTeA Tertile 2 vs. 1	0.825	0.571	1.192	52
Female	Total	PFTeA Tertile 3 vs. 1	0.823	0.637	1.065	
Female	Total	PFTriA Tertile 2 vs. 1	0.894	0.680	1.176	41
Female	Total	PFTriA Tertile 3 vs. 1	0.817	0.583	1.145	
Female	Total	PFUnDA Tertile 2 vs. 1	0.928	0.695	1.238	81
Female	Total	PFUnDA Tertile 3 vs. 1	0.789	0.584	1.066	
Female	Total	PFDS Tertile 2 vs. 1	1.191	0.836	1.698	23
Female	Total	PFDS Tertile 3 vs. 1	1.059	0.803	1.396	
Female	Total	PFHxS Tertile 2 vs. 1	1.183	0.886	1.579	81
Female	Total	PFHxS Tertile 3 vs. 1	0.893	0.735	1.087	
Female	Total	PFOS Tertile 2 vs. 1	1.045	0.818	1.336	72
Female	Total	PFOS Tertile 2 vs. 2	0.709	0.566	0.887	
Female	Total	PFOSA Tertile 2 vs. 1	0.778	0.601	1.007	80
Female	Total	PFOSA Tertile 3 vs. 1	0.673	0.554	0.817	
Female	Total	ΣPFCA Tertile 2 vs. 1	0.696	0.564	0.860	69
Female	Total	ΣPFCA Tertile 3 vs. 1	0.666	0.537	0.825	
Female	Total	ΣPFSA Tertile 2 vs. 1	1.130	0.889	1.435	69
Female	Total	ΣPFSA Tertile 3 vs. 1	0.712	0.587	0.865	
Female	Total	ΣPFAS Tertile 2 vs. 1	1.030	0.800	1.325	72
Female	Total	ΣPFAS Tertile 3 vs. 1	0.740	0.589	0.930	
Male	Free	PFDA Tertile 2 vs. 1	1.445	1.105	1.889	164
Male	Free	PFDA Tertile 3 vs. 1	1.145	0.886	1.480	
Male	Free	PFDoDA Tertile 2 vs. 1	1.415	1.093	1.831	164
Male	Free	PFDoDA Tertile 3 vs. 1	1.017	0.783	1.322	

Male	Free	PFHpA Tertile 2 vs. 1	1.135	0.532	2.421	75
Male	Free	PFHpA Tertile 3 vs. 1	0.816	0.439	1.517	
Male	Free	PFNA Tertile 2 vs. 1	0.983	0.745	1.296	164
Male	Free	PFNA Tertile 3 vs. 1	0.849	0.640	1.128	
Male	Free	PFOA Tertile 2 vs. 1	0.720	0.557	0.930	164
Male	Free	PFOA Tertile 3 vs. 1	0.820	0.623	1.078	
Male	Free	PFTeA Tertile 2 vs. 1	0.448	0.260	0.771	65
Male	Free	PFTeA Tertile 3 vs. 1	0.742	0.568	0.969	
Male	Free	PFTriA Tertile 2 vs. 1	0.769	0.526	1.126	49
Male	Free	PFTriA Tertile 3 vs. 1	0.898	0.558	1.445	
Male	Free	PFUnDA Tertile 2 vs. 1	1.048	0.789	1.392	164
Male	Free	PFUnDA Tertile 3 vs. 1	0.950	0.737	1.224	
Male	Free	PFDS Tertile 2 vs. 1	0.646	0.327	1.275	78
Male	Free	PFDS Tertile 3 vs. 1	1.400	0.806	2.431	
Male	Free	PFHxS Tertile 2 vs. 1	0.745	0.572	0.970	164
Male	Free	PFHxS Tertile 3 vs. 1	0.819	0.617	1.087	
Male	Free	PFOS Tertile 2 vs. 1	0.773	0.591	1.011	150
Male	Free	PFOS Tertile 2 vs. 2	0.799	0.627	1.018	
Male	Free	PFOSA Tertile 2 vs. 1	0.764	0.571	1.023	132
Male	Free	PFOSA Tertile 3 vs. 1	0.813	0.620	1.067	
Male	Free	ΣPFCA Tertile 2 vs. 1	0.718	0.534	0.965	104
Male	Free	ΣPFCA Tertile 3 vs. 1	0.641	0.507	0.812	
Male	Free	ΣPFSA Tertile 2 vs. 1	0.657	0.490	0.880	104
Male	Free	ΣPFSA Tertile 3 vs. 1	0.646	0.512	0.816	
Male	Free	ΣPFAS Tertile 2 vs. 1	0.820	0.622	1.082	150
Male	Free	ΣPFAS Tertile 3 vs. 1	0.840	0.660	1.070	
Male	Bound	PFDA Tertile 2 vs. 1	0.990	0.847	1.157	173
Male	Bound	PFDA Tertile 3 vs. 1	0.796	0.660	0.960	
Male	Bound	PFDoDA Tertile 2 vs. 1	1.237	1.068	1.433	173
Male	Bound	PFDoDA Tertile 3 vs. 1	0.870	0.722	1.049	
Male	Bound	PFHpA Tertile 2 vs. 1	1.006	0.760	1.331	80
Male	Bound	PFHpA Tertile 3 vs. 1	0.782	0.607	1.008	

Male	Bound	PFNA Tertile 2 vs. 1	1.043	0.896	1.215	173
Male	Bound	PFNA Tertile 3 vs. 1	0.719	0.601	0.860	
Male	Bound	PFOA Tertile 2 vs. 1	0.905	0.774	1.059	173
Male	Bound	PFOA Tertile 3 vs. 1	0.696	0.585	0.829	
Male	Bound	PFTeA Tertile 2 vs. 1	0.943	0.673	1.322	68
Male	Bound	PFTeA Tertile 3 vs. 1	1.310	1.032	1.663	
Male	Bound	PFTriA Tertile 2 vs. 1	0.921	0.662	1.282	50
Male	Bound	PFTriA Tertile 3 vs. 1	0.798	0.580	1.097	
Male	Bound	PFUnDA Tertile 2 vs. 1	0.981	0.837	1.150	173
Male	Bound	PFUnDA Tertile 3 vs. 1	0.786	0.658	0.939	
Male	Bound	PFDS Tertile 2 vs. 1	0.947	0.740	1.213	83
Male	Bound	PFDS Tertile 3 vs. 1	0.952	0.740	1.226	
Male	Bound	PFHxS Tertile 2 vs. 1	0.875	0.735	1.040	173
Male	Bound	PFHxS Tertile 3 vs. 1	0.892	0.745	1.067	
Male	Bound	PFOS Tertile 2 vs. 1	0.969	0.809	1.162	158
Male	Bound	PFOS Tertile 2 vs. 2	0.867	0.728	1.031	
Male	Bound	PFOSA Tertile 2 vs. 1	0.929	0.782	1.104	140
Male	Bound	PFOSA Tertile 3 vs. 1	0.701	0.572	0.860	
Male	Bound	ΣPFCA Tertile 2 vs. 1	0.876	0.712	1.078	110
Male	Bound	ΣPFCA Tertile 3 vs. 1	0.730	0.594	0.898	
Male	Bound	ΣPFSA Tertile 2 vs. 1	0.934	0.749	1.166	110
Male	Bound	ΣPFSA Tertile 3 vs. 1	0.874	0.714	1.070	
Male	Bound	ΣPFAS Tertile 2 vs. 1	0.958	0.801	1.147	158
Male	Bound	ΣPFAS Tertile 3 vs. 1	0.880	0.737	1.050	
Male	Total	PFDA Tertile 2 vs. 1	1.351	1.121	1.628	164
Male	Total	PFDA Tertile 3 vs. 1	1.054	0.903	1.230	
Male	Total	PFDoDA Tertile 2 vs. 1	1.382	1.152	1.659	164
Male	Total	PFDoDA Tertile 3 vs. 1	1.020	0.865	1.204	
Male	Total	PFHpA Tertile 2 vs. 1	1.087	0.848	1.393	75
Male	Total	PFHpA Tertile 3 vs. 1	0.873	0.646	1.179	
Male	Total	PFNA Tertile 2 vs. 1	0.978	0.804	1.190	164
Male	Total	PFNA Tertile 3 vs. 1	0.802	0.656	0.981	

Male	Total	PFOA Tertile 2 vs. 1	0.796	0.662	0.958	164
Male	Total	PFOA Tertile 3 vs. 1	0.789	0.648	0.960	
Male	Total	PFTeA Tertile 2 vs. 1	0.573	0.383	0.857	65
Male	Total	PFTeA Tertile 3 vs. 1	0.908	0.729	1.131	
Male	Total	PFTriA Tertile 2 vs. 1	0.788	0.615	1.011	49
Male	Total	PFTriA Tertile 3 vs. 1	0.882	0.628	1.239	
Male	Total	PFUnDA Tertile 2 vs. 1	1.035	0.846	1.266	164
Male	Total	PFUnDA Tertile 3 vs. 1	0.895	0.751	1.067	
Male	Total	PFDS Tertile 2 vs. 1	0.919	0.700	1.207	78
Male	Total	PFDS Tertile 3 vs. 1	1.195	0.941	1.519	
Male	Total	PFHxS Tertile 2 vs. 1	0.780	0.648	0.939	164
Male	Total	PFHxS Tertile 3 vs. 1	0.828	0.672	1.021	
Male	Total	PFOS Tertile 2 vs. 1	0.860	0.715	1.034	150
Male	Total	PFOS Tertile 2 vs. 2	0.819	0.677	0.990	
Male	Total	PFOSA Tertile 2 vs. 1	0.828	0.674	1.018	132
Male	Total	PFOSA Tertile 3 vs. 1	0.804	0.651	0.993	
Male	Total	ΣPFCA Tertile 2 vs. 1	0.777	0.624	0.966	104
Male	Total	ΣPFCA Tertile 3 vs. 1	0.673	0.562	0.807	
Male	Total	ΣPFSA Tertile 2 vs. 1	0.750	0.606	0.927	104
Male	Total	ΣPFSA Tertile 3 vs. 1	0.731	0.592	0.902	
Male	Total	ΣPFAS Tertile 2 vs. 1	0.881	0.728	1.066	150
Male	Total	ΣPFAS Tertile 3 vs. 1	0.849	0.702	1.026	
Juvenile	Free	PFDA Tertile 2 vs. 1	0.959	0.573	1.606	59
Juvenile	Free	PFDA Tertile 3 vs. 1	1.222	0.758	1.969	
Juvenile	Free	PFDoDA Tertile 2 vs. 1	0.967	0.640	1.460	59
Juvenile	Free	PFDoDA Tertile 3 vs. 1	1.123	0.748	1.687	
Juvenile	Free	PFHpA Tertile 2 vs. 1	0.128	0.039	0.416	20
Juvenile	Free	PFHpA Tertile 3 vs. 1	0.855	0.397	1.840	
Juvenile	Free	PFNA Tertile 2 vs. 1	0.864	0.464	1.609	59
Juvenile	Free	PFNA Tertile 3 vs. 1	1.055	0.570	1.953	
Juvenile	Free	PFOA Tertile 2 vs. 1	0.643	0.398	1.039	59
Juvenile	Free	PFOA Tertile 3 vs. 1	0.902	0.574	1.417	

Juvenile	Free	PFTeA Tertile 2 vs. 1	0.132	0.102	0.172	36
Juvenile	Free	PFTeA Tertile 3 vs. 1	0.739	0.514	1.062	
Juvenile	Free	PFTriA Tertile 2 vs. 1	1.077	0.604	1.921	26
Juvenile	Free	PFTriA Tertile 3 vs. 1	1.372	0.759	2.478	
Juvenile	Free	PFUnDA Tertile 2 vs. 1	0.778	0.486	1.246	59
Juvenile	Free	PFUnDA Tertile 3 vs. 1	1.069	0.704	1.624	
Juvenile	Free	PFDS Tertile 2 vs. 1	4.828	1.291	18.053	20
Juvenile	Free	PFDS Tertile 3 vs. 1	7.481	2.522	22.194	
Juvenile	Free	PFHxS Tertile 2 vs. 1	0.644	0.396	1.047	59
Juvenile	Free	PFHxS Tertile 3 vs. 1	0.579	0.385	0.871	
Juvenile	Free	PFOS Tertile 2 vs. 1	0.986	0.611	1.593	58
Juvenile	Free	PFOS Tertile 2 vs. 2	0.752	0.491	1.151	
Juvenile	Free	PFOSA Tertile 2 vs. 1	0.748	0.478	1.170	56
Juvenile	Free	PFOSA Tertile 3 vs. 1	1.055	0.707	1.574	
Juvenile	Free	ΣPFCA Tertile 2 vs. 1	0.929	0.558	1.548	51
Juvenile	Free	ΣPFCA Tertile 3 vs. 1	0.990	0.647	1.515	
Juvenile	Free	ΣPFSA Tertile 2 vs. 1	0.876	0.553	1.387	51
Juvenile	Free	ΣPFSA Tertile 3 vs. 1	0.876	0.437	0.967	
Juvenile	Free	ΣPFAS Tertile 2 vs. 1	1.212	0.729	2.015	58
Juvenile	Free	ΣPFAS Tertile 3 vs. 1	0.913	0.576	1.446	
Juvenile	Bound	PFDA Tertile 2 vs. 1	1.057	0.832	1.344	62
Juvenile	Bound	PFDA Tertile 3 vs. 1	0.532	0.403	0.704	
Juvenile	Bound	PFDoDA Tertile 2 vs. 1	0.889	0.682	1.158	62
Juvenile	Bound	PFDoDA Tertile 3 vs. 1	0.577	0.436	0.764	
Juvenile	Bound	PFHpA Tertile 2 vs. 1	1.102	0.747	1.625	22
Juvenile	Bound	PFHpA Tertile 3 vs. 1	0.933	0.591	1.471	
Juvenile	Bound	PFNA Tertile 2 vs. 1	1.097	0.817	1.472	62
Juvenile	Bound	PFNA Tertile 3 vs. 1	0.601	0.430	0.842	
Juvenile	Bound	PFOA Tertile 2 vs. 1	0.956	0.723	1.264	62
Juvenile	Bound	PFOA Tertile 3 vs. 1	0.562	0.408	0.773	
Juvenile	Bound	PFTeA Tertile 2 vs. 1	1.238	0.989	1.551	37
Juvenile	Bound	PFTeA Tertile 3 vs. 1	1.875	1.379	2.551	

Juvenile	Bound	PFTriA Tertile 2 vs. 1	0.596	0.396	0.896	26
Juvenile	Bound	PFTriA Tertile 3 vs. 1	0.730	0.428	1.243	
Juvenile	Bound	PFUnDA Tertile 2 vs. 1	1.078	0.837	1.388	62
Juvenile	Bound	PFUnDA Tertile 3 vs. 1	0.598	0.450	0.795	
Juvenile	Bound	PFDS Tertile 2 vs. 1	0.765	0.500	1.170	22
Juvenile	Bound	PFDS Tertile 3 vs. 1	0.620	0.397	0.970	
Juvenile	Bound	PFHxS Tertile 2 vs. 1	1.052	0.674	1.641	62
Juvenile	Bound	PFHxS Tertile 3 vs. 1	0.841	0.554	1.275	
Juvenile	Bound	PFOS Tertile 2 vs. 1	1.042	0.730	1.489	61
Juvenile	Bound	PFOS Tertile 2 vs. 2	0.754	0.567	1.003	
Juvenile	Bound	PFOSA Tertile 2 vs. 1	1.056	0.820	1.360	59
Juvenile	Bound	PFOSA Tertile 3 vs. 1	0.510	0.381	0.683	
Juvenile	Bound	ΣPFCA Tertile 2 vs. 1	0.975	0.740	1.285	53
Juvenile	Bound	ΣPFCA Tertile 3 vs. 1	0.524	0.390	0.704	
Juvenile	Bound	ΣPFSA Tertile 2 vs. 1	1.029	0.699	1.517	53
Juvenile	Bound	ΣPFSA Tertile 3 vs. 1	0.761	0.561	1.032	
Juvenile	Bound	ΣPFAS Tertile 2 vs. 1	0.949	0.665	1.354	61
Juvenile	Bound	ΣPFAS Tertile 3 vs. 1	0.728	0.552	0.960	
Juvenile	Total	PFDA Tertile 2 vs. 1	0.990	0.736	1.331	59
Juvenile	Total	PFDA Tertile 3 vs. 1	0.966	0.728	1.282	
Juvenile	Total	PFDoDA Tertile 2 vs. 1	0.930	0.715	1.210	59
Juvenile	Total	PFDoDA Tertile 3 vs. 1	0.943	0.739	1.203	
Juvenile	Total	PFHpA Tertile 2 vs. 1	0.325	0.220	0.479	20
Juvenile	Total	PFHpA Tertile 3 vs. 1	0.902	0.637	1.276	
Juvenile	Total	PFNA Tertile 2 vs. 1	0.955	0.661	1.381	59
Juvenile	Total	PFNA Tertile 3 vs. 1	0.920	0.639	1.324	
Juvenile	Total	PFOA Tertile 2 vs. 1	0.757	0.574	0.998	59
Juvenile	Total	PFOA Tertile 3 vs. 1	0.807	0.625	1.043	
Juvenile	Total	PFTeA Tertile 2 vs. 1	0.295	0.244	0.357	36
Juvenile	Total	PFTeA Tertile 3 vs. 1	0.987	0.761	1.282	
Juvenile	Total	PFTriA Tertile 2 vs. 1	0.929	0.600	1.437	26
Juvenile	Total	PFTriA Tertile 3 vs. 1	1.188	0.792	1.783	

Juvenile	Total	PFUnDA Tertile 2 vs. 1	0.880	0.666	1.163	59
Juvenile	Total	PFUnDA Tertile 3 vs. 1	0.909	0.700	1.180	
Juvenile	Total	PFDS Tertile 2 vs. 1	2.052	1.308	3.217	20
Juvenile	Total	PFDS Tertile 3 vs. 1	2.111	1.517	2.939	
Juvenile	Total	PFHxS Tertile 2 vs. 1	0.784	0.595	1.033	59
Juvenile	Total	PFHxS Tertile 3 vs. 1	0.663	0.524	0.840	
Juvenile	Total	PFOS Tertile 2 vs. 1	1.011	0.771	1.327	58
Juvenile	Total	PFOS Tertile 2 vs. 2	0.766	0.591	0.992	
Juvenile	Total	PFOSA Tertile 2 vs. 1	0.862	0.662	1.122	56
Juvenile	Total	PFOSA Tertile 3 vs. 1	0.885	0.688	1.140	
Juvenile	Total	ΣPFCA Tertile 2 vs. 1	0.936	0.699	1.253	51
Juvenile	Total	ΣPFCA Tertile 3 vs. 1	0.848	0.653	1.100	
Juvenile	Total	ΣPFSA Tertile 2 vs. 1	0.911	0.698	1.191	51
Juvenile	Total	ΣPFSA Tertile 3 vs. 1	0.707	0.550	0.909	
Juvenile	Total	ΣPFAS Tertile 2 vs. 1	1.131	0.838	1.526	58
Juvenile	Total	ΣPFAS Tertile 3 vs. 1	0.863	0.648	1.150	
Adult	Free	PFDA Tertile 2 vs. 1	1.375	1.077	1.755	186
Adult	Free	PFDA Tertile 3 vs. 1	0.960	0.774	1.191	
Adult	Free	PFDoDA Tertile 2 vs. 1	1.216	0.953	1.551	186
Adult	Free	PFDoDA Tertile 3 vs. 1	0.844	0.672	1.062	
Adult	Free	PFHpA Tertile 2 vs. 1	1.770	0.975	3.214	78
Adult	Free	PFHpA Tertile 3 vs. 1	0.745	0.425	1.305	
Adult	Free	PFNA Tertile 2 vs. 1	0.973	0.769	1.232	186
Adult	Free	PFNA Tertile 3 vs. 1	0.716	0.569	0.902	
Adult	Free	PFOA Tertile 2 vs. 1	0.672	0.543	0.833	186
Adult	Free	PFOA Tertile 3 vs. 1	0.680	0.538	0.859	
Adult	Free	PFTeA Tertile 2 vs. 1	0.579	0.402	0.834	81
Adult	Free	PFTeA Tertile 3 vs. 1	0.778	0.608	0.994	
Adult	Free	PFTriA Tertile 2 vs. 1	0.831	0.619	1.114	64
Adult	Free	PFTriA Tertile 3 vs. 1	0.701	0.446	1.102	
Adult	Free	PFUnDA Tertile 2 vs. 1	1.108	0.858	1.430	186
Adult	Free	PFUnDA Tertile 3 vs. 1	0.899	0.701	1.152	

Adult	Free	PFDS Tertile 2 vs. 1	0.566	0.307	1.043	81
Adult	Free	PFDS Tertile 3 vs. 1	1.085	0.650	1.814	
Adult	Free	PFHxS Tertile 2 vs. 1	0.863	0.679	1.097	186
Adult	Free	PFHxS Tertile 3 vs. 1	0.880	0.693	1.116	
Adult	Free	PFOS Tertile 2 vs. 1	0.885	0.692	1.131	164
Adult	Free	PFOS Tertile 2 vs. 2	0.746	0.596	0.935	
Adult	Free	PFOSA Tertile 2 vs. 1	0.717	0.551	0.935	156
Adult	Free	PFOSA Tertile 3 vs. 1	0.698	0.563	0.864	
Adult	Free	ΣPFCA Tertile 2 vs. 1	0.653	0.514	0.829	122
Adult	Free	ΣPFCA Tertile 3 vs. 1	0.581	0.475	0.711	
Adult	Free	ΣPFSA Tertile 2 vs. 1	0.808	0.622	1.048	122
Adult	Free	ΣPFSA Tertile 3 vs. 1	0.641	0.519	0.792	
Adult	Free	ΣPFAS Tertile 2 vs. 1	0.863	0.675	1.105	164
Adult	Free	ΣPFAS Tertile 3 vs. 1	0.770	0.616	0.962	
Adult	Bound	PFDA Tertile 2 vs. 1	1.008	0.856	1.187	192
Adult	Bound	PFDA Tertile 3 vs. 1	0.722	0.598	0.872	
Adult	Bound	PFDoDA Tertile 2 vs. 1	1.290	1.093	1.523	192
Adult	Bound	PFDoDA Tertile 3 vs. 1	0.882	0.732	1.064	
Adult	Bound	PFHpA Tertile 2 vs. 1	1.060	0.793	1.416	81
Adult	Bound	PFHpA Tertile 3 vs. 1	0.730	0.571	0.934	
Adult	Bound	PFNA Tertile 2 vs. 1	0.887	0.746	1.055	192
Adult	Bound	PFNA Tertile 3 vs. 1	0.699	0.586	0.834	
Adult	Bound	PFOA Tertile 2 vs. 1	0.866	0.726	1.033	192
Adult	Bound	PFOA Tertile 3 vs. 1	0.732	0.612	0.876	
Adult	Bound	PFTeA Tertile 2 vs. 1	0.896	0.679	1.183	83
Adult	Bound	PFTeA Tertile 3 vs. 1	0.833	0.651	1.066	
Adult	Bound	PFTriA Tertile 2 vs. 1	0.788	0.594	1.045	65
Adult	Bound	PFTriA Tertile 3 vs. 1	0.738	0.571	0.955	
Adult	Bound	PFUnDA Tertile 2 vs. 1	0.929	0.783	1.102	192
Adult	Bound	PFUnDA Tertile 3 vs. 1	0.742	0.615	0.895	
Adult	Bound	PFDS Tertile 2 vs. 1	0.974	0.765	1.240	84
Adult	Bound	PFDS Tertile 3 vs. 1	0.823	0.630	1.074	

Adult	Bound	PFHxS Tertile 2 vs. 1	0.932	0.780	1.114	192
Adult	Bound	PFHxS Tertile 3 vs. 1	0.999	0.827	1.205	
Adult	Bound	PFOS Tertile 2 vs. 1	0.851	0.700	1.034	169
Adult	Bound	PFOS Tertile 2 vs. 2	0.864	0.711	1.048	
Adult	Bound	PFOSA Tertile 2 vs. 1	0.910	0.760	1.090	161
Adult	Bound	PFOSA Tertile 3 vs. 1	0.652	0.531	0.801	
Adult	Bound	ΣPFCA Tertile 2 vs. 1	0.803	0.638	1.009	126
Adult	Bound	ΣPFCA Tertile 3 vs. 1	0.720	0.578	0.898	
Adult	Bound	ΣPFSA Tertile 2 vs. 1	0.978	0.779	1.227	126
Adult	Bound	ΣPFSA Tertile 3 vs. 1	0.969	0.760	1.236	
Adult	Bound	ΣPFAS Tertile 2 vs. 1	0.874	0.720	1.060	169
Adult	Bound	ΣPFAS Tertile 3 vs. 1	0.865	0.712	1.052	
Adult	Total	PFDA Tertile 2 vs. 1	1.320	1.113	1.566	186
Adult	Total	PFDA Tertile 3 vs. 1	0.907	0.791	1.040	
Adult	Total	PFDoDA Tertile 2 vs. 1	1.266	1.060	1.513	186
Adult	Total	PFDoDA Tertile 3 vs. 1	0.892	0.759	1.048	
Adult	Total	PFHpA Tertile 2 vs. 1	1.306	1.047	1.627	78
Adult	Total	PFHpA Tertile 3 vs. 1	0.767	0.586	1.004	
Adult	Total	PFNA Tertile 2 vs. 1	0.949	0.797	1.129	186
Adult	Total	PFNA Tertile 3 vs. 1	0.709	0.600	0.838	
Adult	Total	PFOA Tertile 2 vs. 1	0.756	0.641	0.892	186
Adult	Total	PFOA Tertile 3 vs. 1	0.710	0.592	0.851	
Adult	Total	PFTeA Tertile 2 vs. 1	0.701	0.524	0.939	81
Adult	Total	PFTeA Tertile 3 vs. 1	0.827	0.661	1.035	
Adult	Total	PFTriA Tertile 2 vs. 1	0.805	0.663	0.979	64
Adult	Total	PFTriA Tertile 3 vs. 1	0.758	0.545	1.054	
Adult	Total	PFUnDA Tertile 2 vs. 1	1.050	0.867	1.271	186
Adult	Total	PFUnDA Tertile 3 vs. 1	0.847	0.702	1.023	
Adult	Total	PFDS Tertile 2 vs. 1	0.906	0.703	1.168	81
Adult	Total	PFDS Tertile 3 vs. 1	1.016	0.781	1.322	
Adult	Total	PFHxS Tertile 2 vs. 1	0.873	0.730	1.044	186
Adult	Total	PFHxS Tertile 3 vs. 1	0.907	0.751	1.095	

Adult	Total	PFOS Tertile 2 vs. 1	0.897	0.746	1.078	164
Adult	Total	PFOS Tertile 2 vs. 2	0.781	0.648	0.940	
Adult	Total	PFOSA Tertile 2 vs. 1	0.796	0.655	0.967	156
Adult	Total	PFOSA Tertile 3 vs. 1	0.705	0.588	0.846	
Adult	Total	ΣPFCA Tertile 2 vs. 1	0.714	0.587	0.869	122
Adult	Total	ΣPFCA Tertile 3 vs. 1	0.618	0.587	0.725	
Adult	Total	ΣPFSA Tertile 2 vs. 1	0.856	0.698	1.051	122
Adult	Total	ΣPFSA Tertile 3 vs. 1	0.728	0.593	0.893	
Adult	Total	ΣPFAS Tertile 2 vs. 1	0.885	0.736	1.065	164
Adult	Total	ΣPFAS Tertile 3 vs. 1	0.799	0.665	0.960	
